# Single nucleotide and copy number variants of cancer driver genes inform drug response in multiple cancers

**DOI:** 10.1371/journal.pone.0306343

**Published:** 2024-07-31

**Authors:** Zeyuan Wang, Hong Gu, Pan Qin, Jia Wang

**Affiliations:** 1 School of Control Science and Engineering, Dalian University of Technology, Dalian, Liaoning, China; 2 Department of Breast Surgery, Institute of Breast Disease, Second Hospital of Dalian Medical University, Dalian, Liaoning, China; University of Alabama at Birmingham, UNITED STATES

## Abstract

Due to the heterogeneity of cancer, precision medicine has been a major challenge for cancer treatment. Determining medication regimens based on patient genotypes has become a research hotspot in cancer genomics. In this study, we aim to identify key biomarkers for targeted therapies based on single nucleotide variants (SNVs) and copy number variants (CNVs) of genes. The experiment is carried out on 7 cancers on the Encyclopedia of Cancer Cell Lines (CCLE) dataset. Considering the high mutability of driver genes which result in abundant mutated samples, the effect of data sparsity can be eliminated to a large extent. Therefore, we focus on discovering the relationship between driver mutation patterns and three measures of drug response, namely area under the curve (AUC), half maximal effective concentration (EC50), and log2-fold change (LFC). First, multiple statistical methods are applied to assess the significance of difference in drug response between sample groups. Next, for each driver gene, we analyze the extent to which its mutations can affect drug response. Based on the results of multiple hypothesis tests and correlation analyses, our main findings include the validation of several known drug response biomarkers such as *BRAF*, *NRAS*, *MAP2K1*, *MAP2K2*, and *CDKN2A*, as well as genes with huge potential to infer drug responses. It is worth emphasizing that we identify a list of genes including *SALL4*, *B2M*, *BAP1*, *CCDC6*, *ERBB4*, *FOXA1*, *GRIN2A*, and *PTPRT*, whose impact on drug response spans multiple cancers and should be prioritized as key biomarkers for targeted therapies. Furthermore, based on the statistical p-values and correlation coefficients, we construct gene-drug sensitivity maps for cancer drug recommendation. In this work, we show that driver mutation patterns could be used to tailor therapeutics for precision medicine.

## Introduction

Precision medicine for cancer has been challenging due to chromosomal and genetic instability [1–4]. Considering the wide range and serious side effects of anticancer drugs, it is critical to implement customized therapies for the sake of patients. Over the past few decades, several large public cancer datasets such as the Cancer Cell Lines Encyclopedia (CCLE) [5] and the Genomics of Drug Sensitivity in Cancer (GDSC) [6] have emerged, which gave rise to a variety of genetics-based methods for drug response prediction [7–10]. However, due to the large number of genomic features and a relatively small sample size, lack of interpretability and low robustness are the main problems faced by existing models. So far, biomarker-driven approaches for drug recommendation that can be used clinically have not been fully developed.

According to recent studies, genetic mutations is a key factor that lead to the differences in drug response [11, 12]. Thus, the search for drug targets which drive the development of drug sensitivity or resistance from massive data can improve the effectiveness of cancer treatment. Previous researches have shown that drug sensitivity is heterogeneous [13]. Lee et al. studied on 19 non-small cell lung cancer patients and identified *EGFR* T790M as the most common mechanism of resistance. However, *TP53* predominated in the T790M-negative tumors [14]. In addition, Van et al. studied 45 *BRAF* V600-mutant metastatic melanoma patients with vemurafenib or dabrafenib resistance. They found that 44.4% resistance were caused by alterations in *MAPK* pathway or downstream effectors. The changes of *PI3K* pathway and *HOXD8* or *RAC1* lead to the resistance of the remaining patients [15]. Besides single nucleotide variants (SNVs), researchers also found that characterization of copy number variants (CNVs) might inform drug response and future precision medicine clinical trials. Filipe et al. studied on 21 high-grade serous ovarian carcinoma (HGSOC) samples and aimed to reveal the relationship between somatic CNVs of driver genes and drug sensitivity. They identified five prevalent CNVs (*MYC*, *PIK3CA*, *CCNE1*, *KRAS* and *TERT*) from multi-regional HGSOC data and reason that their strong selection should prioritize them as key biomarkers for targeted therapies [16].

Based on the results of clinical trials, variations associated with driver pathways and driver gene sets have potential relationships with drug response. However, due to insufficient sample size, the existing findings still need to be further confirmed. On the other hand, current drug sensitivity prediction methods based on feature selection and machine learning face the problems of overfitting and model instability [17]. To this end, we aim to explore key biomarkers for targeted therapies based on the SNVs and CNVs of driver genes. Considering that the mutation rates in driver genes are much higher than passengers, focusing on driver gene sets can effectively avoid data sparseness, which leads to a more convincing result.

**Fig 1:**
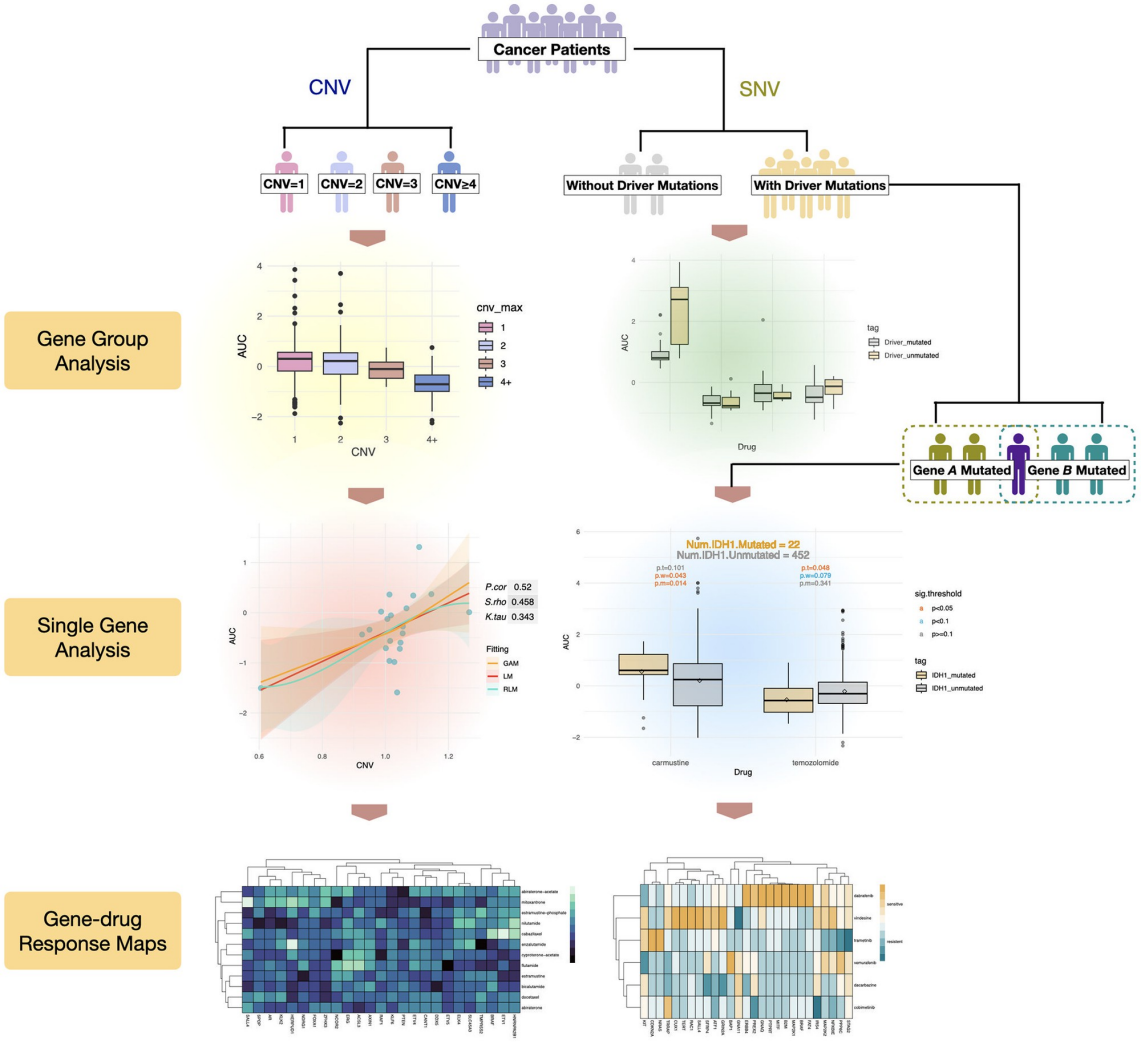
Diagram summarizing the research workflow and main results.

In this work, we use multiple cancer drug response data from the CCLE dataset to analyze the impact of gene mutation patterns on drug response from different aspects. The research framework is shown in Fig 1. By taking three measures of drug response into consideration, we first implement group analyses for SNV and CNV to assess the variance in drug response between the two populations. Next, we conduct single gene-drug response analyses to explore whether specific driver genes influence drug response more than others and which genetic mutations can serve as biomarkers of drug sensitivity. Based on the statistical p-values and correlation coefficients, we construct gene-drug sensitivity maps which integrate the impact of driver gene mutations on drug sensitivity. In addition, we segment the samples by cancer type and further examine the relationship between genetic mutations and drug response. Based on the statistical results, we find a bunch of potential biomarkers for drug sensitivity prediction. Finally, the validation using the GDSC dataset increases the credibility of our results to a huge extent. Our findings promote the revelations of the molecular mechanism and heterogeneity of drug response.

## Materials and methods

### Data collection

In this study, we use 4 datasets from CCLE, including two drug screen datasets and two corresponding genomic characterization datasets. The first drug screen dataset is the secondary PRISM Repurposing 19Q4 dataset, which contains the results of pooled-cell line chemical-perturbation viability screens for 1,448 compounds screened against 499 cell lines in a 8-step, 4-fold dilution, starting from 10*μ*M. In this dataset, we choose two measures to represent drug response, which are half maximal effective concentration (EC50) and area under the curve (AUC). Another widely used measure, namely half-maximal inhibitory concentration (IC50), is not included due to redundant missing values. The other drug dataset is the PRISM Repurposing Public 23Q2 dataset, which is the newest public cell line collection. In this dataset, 1,514 compounds are screened at a dose of 2.5 *μ*M with a 5-day treatment against 906 cancer cell lines. Since the dose applied is constant, a log2-fold change (LFC) relative to negative-control wells is used to measure drug response.

Correspondingly, the two genomic characterization datasets are selected for joint analysis, which are the DepMap Public 19Q4 and 23Q2 datasets. We use SNV and CNV as gene features, which are respectively binary and real-valued variables. The putative driver genes are identified based on the Cancer Gene Census (CGC) of Somatic Mutations in Cancer (COSMIC) [18]. In this study, we focus on 7 types of cancers, which are breast cancer (BRCA), non-small cell lung cancer (NSCLC), colorectal cancer (CRC), prostate cancer (PRAD), melanoma (SKCM), chronic myelogenous leukemia (CML), and glioblastoma (GB). For each cancer type, the corresponding drugs and driver genes shown in Table 1 are included in our analysis.

**Table 1:** Details of drug names, driver gene sets, and drug response measures for each cancer.

Cancer	Drugs	CGC driver genes	Measures
Breast cancer (BRCA)	fulvestrant, doxorubicin, ixabepilone, docetaxel, capecitabine, paclitaxel, aminoglutethimide, carmofur, everolimus, palbociclib, cyclophosphamide, exemestane, epirubicin, raloxifene, anastrozole, 5-fluorouracil, formestane, lapatinib, toremifene, letrozole, abemaciclib, gemcitabine, tamoxifen, ribociclib, vindesine	*AKT1*, *ARID1A*, *ARID1B*, *BAP1*, *BARD1*, *BRCA2*, *CASP8*, *CCND1*, *CDH1*, *CDKN1B*, *CTCF*, *EP300*, *ERBB2*, *ESR1*, *ETV6*, *FOXA1*, *GATA3*, *IKZF3*, *IRS4*, *KEAP1*, *MAP2K4*, *MAP3K1*, *MAP3K13*, *NCOR1*, *NOTCH1*, *NTRK3*, *PBRM1*, *PIK3CA*, *PPM1D*, *RB1*, *SALL4*, *TBX3*, *TP53*	AUC, EC50, LFC
Non-small cell lung cancer (NSCLC)	erlotinib, docetaxel, etoposide, vinorelbine, paclitaxel, alectinib, afatinib, gefitinib, crizotinib, osimertinib, gemcitabine, icotinib, brigatinib, vindesine	*AKT1*, *ALK*, *BAP1*, *BRAF*, *CCDC6*, *CD74*, *DDR2*, *DROSHA*, *EGFR*, *EML4*, *ERBB2*, *ERBB4*, *EZR*, *FGFR2*, *HIP1*, *KDR*, *KEAP1*, *KIF5B*, *LRIG3*, *MAP2K1*, *MAP2K2*, *NFE2L2*, *NKX2–1*, *NRG1*, *PIK3CB*, *PTPN13*, *RET*, *ROS1*, *SDC4*, *SLC34A2*, *SMARCA4*, *SOX2*, *STK11*, *TFG*, *TPM3*, *TPR*	AUC, EC50, LFC
Colorectal cancer (CRC)	floxuridine, regorafenib, capecitabine, carmofur, 5-fluorouracil, SN-38, ftorafur, doxifluridine, tipiracil, irinotecan, oxaliplatin	*AKT1*, *APC*, *AXIN1*, *AXIN2*, *B2M*, *BAX*, *BCL9L*, *BRAF*, *CTNNB1*, *CUX1*, *EIF3E*, *EP300*, *FBXW7*, *GRIN2A*, *HIF1A*, *IKZF3*, *KRAS*, *MAP2K1*, *MAP2K4*, *MDM2*, *MLH1*, *MSH2*, *MSH6*, *NTRK2*, *PIK3CA*, *PIK3R1*, *POLE*, *PTPRK*, *PTPRT*, *QKI*, *RAD21*, *RSPO2*, *RSPO3*, *SALL4*, *SFRP4*, *SMAD2*, *SMAD3*, *SMAD4*, *SRC*, *TBL1XR1*, *TCF7L2*, *TGFBR2*, *TP53*, *UBR5*	AUC, EC50, LFC
Prostate cancer (PRAD)	estramustine-phosphate, estramustine, nilutamide, bicalutamide, docetaxel, cabazitaxel, flutamide, abiraterone-acetate, mitoxantrone, enzalutamide, cyproterone-acetate, abiraterone	*ACSL3*, *AR*, *AXIN1*, *BRAF*, *CANT1*, *DDX5*, *ELK4*, *ERG*, *ETV1*, *ETV4*, *ETV5*, *FOXA1*, *HERPUD1*, *HNRNPA2B1*, *KLF6*, *KLK2*, *NCOR2*, *NDRG1*, *PTEN*, *RAF1*, *SALL4*, *SLC45A3*, *SPOP*, *TMPRSS2*, *ZFHX3*	AUC, EC50
Melanoma (SKCM)	dabrafenib, vemurafenib, dacarbazine, trametinib, cobimetinib, vindesine	*ATF1*, *B2M*, *BAP1*, *BRAF*, *CDKN2A*, *CUX1*, *ERBB4*, *FAT4*, *GNA11*, *GNAQ*, *GRIN2A*, *IRS4*, *KIT*, *MAP2K1*, *MAP2K2*, *MITF*, *NFKBIE*, *NRAS*, *PPP6C*, *PREX2*, *PTPRT*, *RAC1*, *SALL4*, *SFRP4*, *STAG2*, *TERT*, *TRRAP*	AUC, EC50, LFC
Chronic myelogenous leukemia (CML)	bosutinib, hydroxyurea, busulfan, dasatinib, decitabine, cyclophosphamide, homoharringtonine, ponatinib, imatinib, nilotinib, mechlorethamine	*ABL1*, *BCR*, *CCDC6*, *CSF3R*, *ETNK1*, *GATA2*, *HOXA11*, *JAK2*, *MECOM*, *MSI2*, *PCM1*, *PDGFRB*, *RPL22*, *SETBP1*	AUC, EC50
Glioblastoma (GB)	carmustine, temozolomide	*DAXX*, *GOPC*, *HIF1A*, *IDH1*, *IDH2*, *LZTR1*, *MDM4*, *PDGFRA*, *PIK3CA*, *PIK3R1*, *ROS1*, *SALL4*, *STAG2*, *TERT*	AUC, EC50

We also include the GDSC2 dataset from GDSC for validation, which includes 939 samples. In GDSC2, two drug response measures AUC and IC50 are available. The gene SNV data of pan-cancer samples are used as features.

### Statistical and analytical methods

Based on the gene mutation pattern and drug response data, we perform a series of statistical analysis to reveal their relationship, including drug response distribution analysis, gene group analysis, single gene analysis, and cancer-specific sample analysis. The experiments on gene SNV and CNV are implemented separately.

#### Distribution and correlation analysis of drug response measures

**Fig 2:**
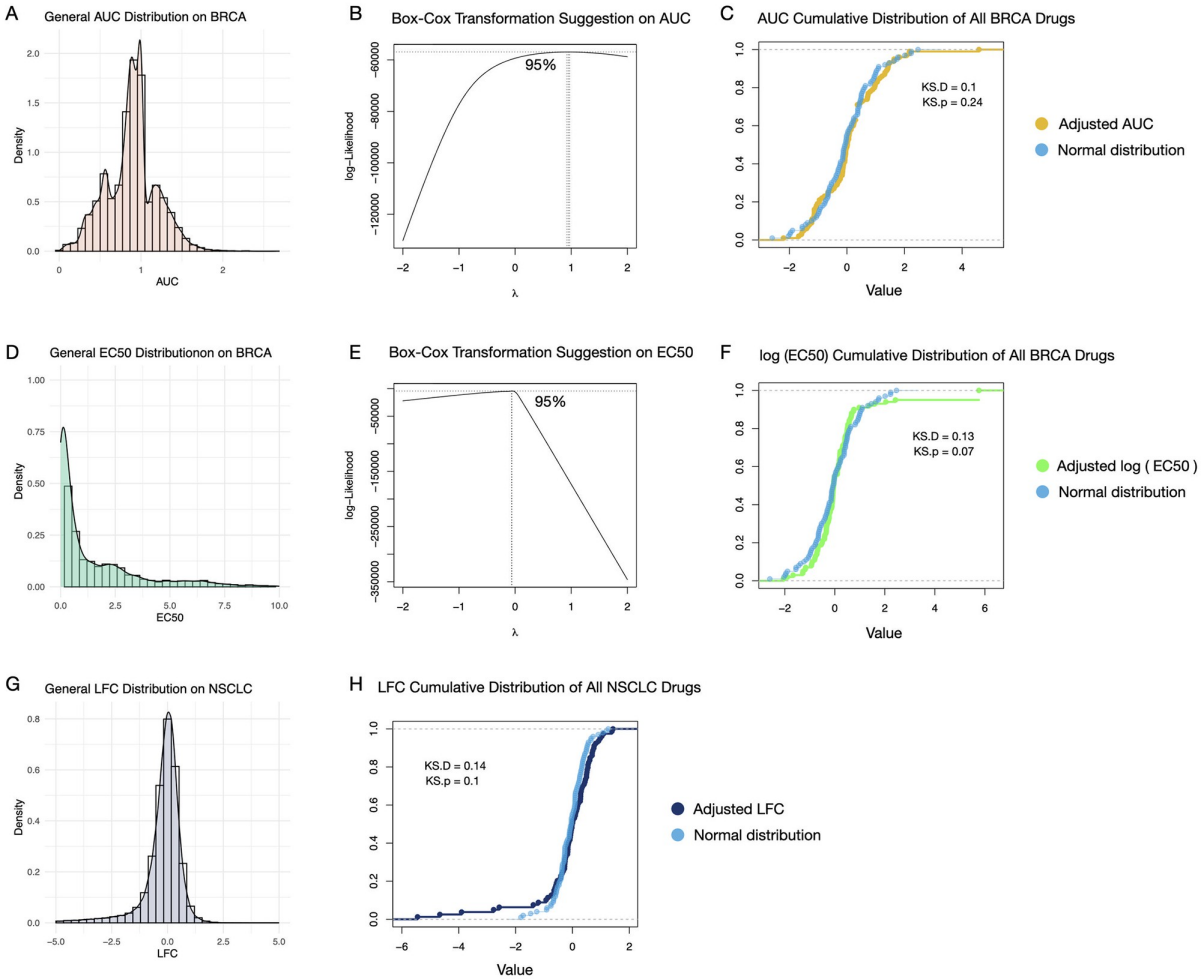
Distributions and transformations of drug response measures. A: Density plots of BRCA drug AUCs. B: Box-Cox transformation suggestion on AUC. C: Cumulative distribution of adjusted AUC and a randomly generated normal distribution. D: Density plots of BRCA drug EC50s. E: Box-Cox transformation suggestion on EC50. F: Cumulative distribution of adjusted EC50 and a randomly generated normal distribution. G: Density plots of NSCLC drug LFCs. H: Cumulative distribution of adjusted LFC and a randomly generated normal distribution.

Considering a normal distribution is optimal for difference significance analysis, especially for the Welch t-tests [19], we first assess the normality of the distribution of all drug response measures. The histograms and density plots of merged AUC, EC50, and LFC on multiple drugs are shown in Fig 2A, 2D and 2G, respectively. It can be observed that AUC and LFC are close to a normal distribution, while the distribution of EC50 lacks normality. Next, we apply Box-Cox transformations [20] for AUC and EC50, which contain only non-negative values. According to Fig 2B and 2E, the estimated λ is close to 1 for AUC, indicating that the original AUC values show sufficient normality. The estimated λ for EC50 is close to 0, which means a log transformation should be imposed for EC50. For LFC, which contains negative values, we apply a Yeo-Johnson transformation [21] to increase normality. The transformed drug response measures are then adjusted by the mean values so that the new mean values become 0. In Fig 2C, 2F and 2H, the cumulative distributions of the processed AUC, EC50, and LFC values are shown and compared with 100 points generated randomly from a normal distribution. An asymptotic one-sample Kolmogorov-Smirnov test [22] is done to measure data normality, where KS.D represents the absolute max distance between the cumulative distributions of the adjusted values and fitted normal distribution. By setting the significance level of the p-value of Kolmogorov-Smirnov test, namely KS.p, to 0.05, results show that the distributions of all three transformed drug measures are not statistically different from the normal distribution.

We further calculate the correlation coefficients between drug AUC and EC50 to evaluate the substitutability of these two measures. In S1 Fig, three correlations are assessed for each type of cancer drugs, namely, Pearson’s correlation (P.cor), Spearman’s rho (S.rho), and Kendall’s tau (K.tau). In addition, we use three fitting models for capturing linear or non-linear relationships, which are linear regression model (LM), robust linear model (RLM), and generalized additive model (GAM). The correlations and fittings are calculated based on the *stats* R package. In S1A–S1F Fig, the vertical lines converged by points in the scatter plots and the correlation coefficients reveal a weak correlation between the two measures. Therefore, we suppose that although AUC and EC50 have commonalities in measuring drug sensitivity, it is necessary to analyze the two measures separately.

#### Analyzing the role of driver SNVs in affecting drug response

Our main purpose is to investigate whether and to what extent driver events can affect drug response. First, we focus on examining SNVs in driver gene sets. For each type of cancer, the samples are divided into groups with driver SNVs and groups without driver SNVs based on the CGC driver list, where samples with at least one driver gene mutation are classified to the first group. Next, for each drug, we use three statistical tests to assess if the location shift of drug response between two populations is different from 0. Following common choices, 0.01, 0.05 and 0.1 are used as the thresholds of p-values to measure the significance of difference, representing a strong, moderate, and weak evidence, separately [23, 24]. Based on the normality of preprocessed drug measures, the two-sample Welch t-test (*t.test* function of the *stats* R package) is appropriate for the analysis. In addition, considering that the drug responses in certain groups may not conform to the normal distribution due to small sample sizes, the Mann-Whitney-Wilcoxon test (*wilcox.test* function of the *stats* R package) and the Mood’s median test (*median_test* function of the *coin* R package) are also used to ensure the robustness of the results. The distribution of drug responses between the two populations are shown by box plots.

Furthermore, we analyze the impact of the SNVs of each driver gene on drug response to discover drug sensitivity biomarkers. Based on the results of statistical tests, the drug sensitivity SNV biomarkers are identified when all three p-values of the gene are less than 0.05, or at least one p-value is less than 0.01. The importance of the resulting biomarkers is evaluated from three perspectives, which are level of significance (to what extent can gene SNV affect a specific drug response), range of drugs (how many drugs of a specific cancer can be affected), and range of diseases (how many cancers the drug response of which can be affected), respectively.

#### Discovering the associations between drug response and driver CNVs

In order to discover the relationship between driver CNVs and drug response, we use a framework similar to the previous one for SNV which includes group analysis and single gene analysis. However, considering the difference in data formats of CNV and SNV, we apply different analytical methods. First, we visualize and examine the distributions of driver CNVs. It can be seen from S2 Fig that the CNV values follow a normal distribution with mean 1. Next, we adjust the real-valued CNVs into integers and classify the samples into groups with 1, 2, 3, and 4+ maximum driver CNVs. We then use box plots to show the location shift among groups. For single gene CNV, we implement the same approach as examining the relationship between drug response measures, including three correlations (P.cor, S.rho, K.tau) and three fitting models (LM, RLM, GAM). Therefore, the linear or non-linear relationships between CNVs and drug response can be fully discovered.

#### Construction of gene-drug sensitivity maps for drug recommendation

Based on the previous analytical results, we provide a series of gene-drug sensitivity maps generated by the *pheatmap* function of the *pheatmap* R package for drug recommendation. For drug EC50, considering that a lower response value means the drug is more sensitive while a smaller p-value indicates a more significant difference between groups, we first set the t-test p-value of a gene to negative when the mean EC50 value of the unmutated group is lower than that of the mutated group. In this case, the SNV of the gene reduces drug sensitivity. In the other case where the mean EC50 value of the mutated group is lower, the p-value stays the same. For drug AUC, since a higher value indicates the drug is more sensitive, the p-values are treated in the opposite way. Next, we take the reciprocal of the p-values to show significance in maps. On the other hand, we use the Pearson correlations between continuous CNV values and drug response to generate the maps where positive or negative values of the correlation coefficient indicate sensitivity or inhibition as the copy number increases. In the gene-drug sensitivity maps, the genes and drugs are hierarchically clustered, from which further relationships among them can be inferred.

## Results

### Driver SNVs affect drug response in multiple cancers

**Fig 3:**
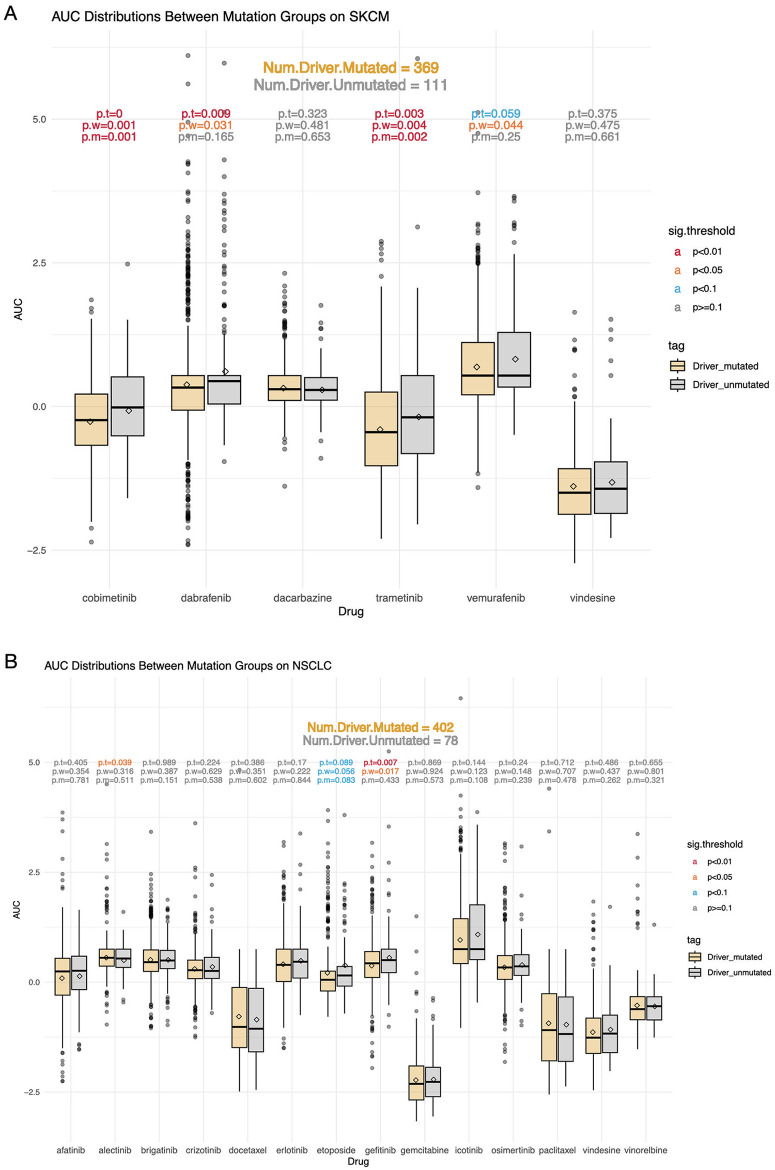
Distribution differences of drug responses between mutation groups. A: Box plots showing AUC distributions of SKCM drugs with or without SNVs of SKCM driver genes. B: Box plots showing AUC distributions of NSCLC drugs with or without SNVs of NSCLC driver genes. The diamond shapes represent mean values.

By dividing samples with driver gene mutations into one group and the rest into the other group, we examine the distribution of responses to each drug. Fig 3 shows the results for drug AUC on the SKCM and NSCLC datasets. For SKCM, the number of samples with and without driver SNVs are 369 and 111, respectively. As shown in Fig 3A, among the 6 drugs of SKCM, the AUC of cobimetinib, trametinib, dabrafenib, and vemurafenib show statistical differences between the two groups by at least two methods. Specifically, cobimetinib and trametinib reach the highest significance level by all statistical methods, indicating that driver SNVs play an important role in affecting their efficacies. For NSCLC, the number of samples with and without driver SNVs are 402 and 78, respectively. Fig 3B shows that two out of 14 NSCLC drugs meet the significance threshold, which are gefitinib and etoposide. In general, we find that the impact of SNVs in driver gene sets on drug response varies by drug. In addition, by comparing the mean AUC values of the drugs between the two populations, it can be seen that for drugs showing statistical differences, the mean AUC values are overall lower in the population with driver SNVs.

On the other hand, S3 Fig indicates that the general impact of driver gene mutations on drug EC50 is lower than AUC. As shown in S3A Fig, the only SKCM drug that meets the 0.05 p-value threshold by at least two statistical methods is cobimetinib. According to S3B Fig, for NSCLC, gefitinib is the only drug showing statistical difference between the two groups with the p-value of t-test being 0.071. We further evaluate the relationship among the p-values resulting from different statistical methods by their ranks, the results for SKCM and NSCLC drugs are summarized as S4A–S4F and S5A–S5F Figs, respectively. The linear fitting results show that, for SKCM drugs, which contain more significant drugs compared to NSCLC, the p-values of the three statistical methods show higher consistency. According to S4G–S4I Fig, we also discover that the ranking of drug p-values show a linear relationship between AUC and EC50 for SKCM drugs. A similar relationship exists in the t-test p-value ranks for NSCLC drugs, as shown in S5G Fig. Such finding indicates that although the differences of drug EC50 are less significant, the impact of driver gene SNVs on drug AUC and EC50 are consistent to a large extent.

**Fig 4:**
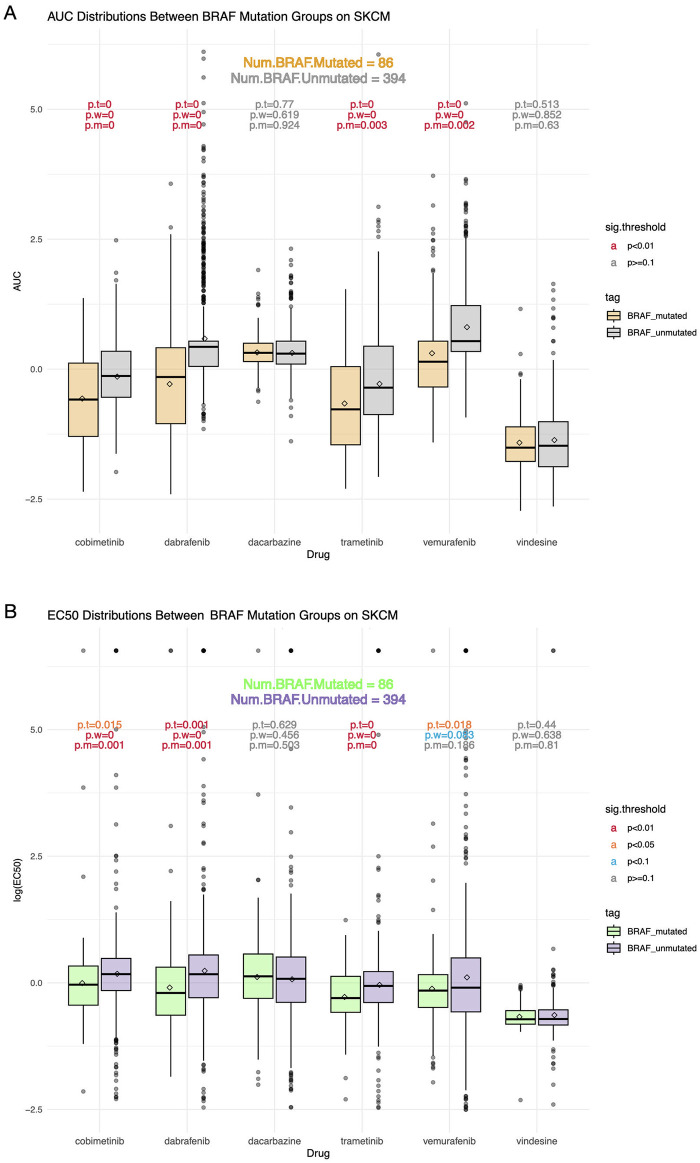
Summary of *BRAF* mutations affecting SKCM drug responses. A: Box plots showing AUC distributions of SKCM drugs with or without SNVs of *BRAF*. B: Box plots showing EC50 distributions of SKCM drugs with or without SNVs of *BRAF*.

Next, we explore the relationship between individual driver SNVs and drug response. For each of the 28 driver genes of SKCM, the samples are divided into two populations based on whether it is mutated or not. Taking gene *BRAF* as an example, the AUC distributions of SKCM drugs are shown in Fig 4A. The number of samples with and without *BRAF* SNVs are 86 and 394, respectively. We find that similar to the previous result, the 4 drugs cobimetinib, trametinib, dabrafenib, and vemurafenib show statistical differences between the two groups. Moreover, since there is no interference from other genes, the p-values based on all methods are closer to 0. For the above four drugs, it can be seen that samples with *BRAF* mutations have an overall lower AUC than those without *BRAF* mutations. However, for vindesine and dacarbazine, *BRAF* SNVs do not have a significant impact on drug AUC. In addition, according to Fig 4B, the significance of drug EC50 are consistent with AUC, where the EC50 distribution of the afore-mentioned 4 drugs are statistically different tested by at least two methods. The inference of *BRAF* being a biomarker of drug sensitivity has been verified biologically. According to the OncoKB database [25], *BRAF* serves as a biomarker predictive of drug response with the highest level of evidence. For all 28 SKCM driver genes, the SNVs of which have a significant impact on the AUC of more than one drug are *B2M*, *BAP1*, *FAT4*, *MAP2K1*, *PTPRT*, *SALL4*, *STAG2*, *TERT*, *CDKN2A*, *GNAQ*, *GRIN2A*, *MITF*, *NRAS*, and *BRAF*. For drug EC50, the identified predictive genes are *BAP1*, *CDKN2A*, *ERBB4*, *GNAQ*, *MAP2K2*, *MITF*, *RAC1*, *TERT*, *NRAS*, and *BRAF*. Among them, *BRAF*, *NRAS*, *MAP2K1*, *MAP2K2*, and *CDKN2A* has been biologically verified as drug sensitivity biomarkers according to OncoKB, which provide solid support for the confidence of our results. S6–S9 Figs show the box plots of *NRAS*, *GRIN2A*, *CDKN2A*, and *FAT4*.

We then apply the same methods on BRCA, NSCLC, CRC, PRAD, CML, and GB. Table 2 summarizes the impact of each driver SNV on the response to each drug, where the number of drugs whose sensitivity can be significantly affected by driver gene mutations are categorized into {1, 2, 3+}. For example, *BRAF* SNV can affect both AUC and EC50 of 4 drugs of SKCM, so it is classified into the 3+ category for both measures. In Table 2, we annotate the genes that affect both drug AUC and EC50 (underlined), as well as the confirmed drug response biomarkers included in OncoKB (in bold). We find that despite the lack of strong correlation between AUC and EC50, more than half of the resulting genes are able to affect AUC and EC50 simultaneously. In addition, apart from the already confirmed drug response biomarkers, a number of new biomarkers are identified with a high confidence. On the other hand, the number of significant genes and their distributions across categories vary greatly across cancer drugs, mainly due to the differences in driver gene sets and the number of drugs. However, we find a list of genes showing significance across multiple cancers. Among them, *BRAF* and *SALL4* affect the drug response across 4 cancers. *MAP2K1* show significance across three cancers. *AKT1*, *B2M*, *BAP1*, *CCDC6*, *ERBB2*, *ERBB4*, *FOXA1*, *GRIN2A*, *MAP2K2*, *PIK3CA*, *PTPRT*, and *TP53* are significant across two cancers. Thus, we infer that the identified genes not only plays an important role in the development of multiple cancers, but also affects the response of various cancer drugs.

**Table 2:** Significant driver genes predictive of drug response by SNV. Underlined genes represent they can affect both drug AUC and EC50. Genes in bold represent confirmed drug response biomarkers included in OncoKB.

Cancer	Drug sensitivity biomarkers for AUC			Drug sensitivity biomarkers for EC50		
	For 1 drug	For 2 drugs	For 3+ drugs	For 1 drug	For 2 drugs	For 3+ drugs
BRCA	***AKT1***, *CTCF*, *GATA3*, *IRS4*, *MAP3K1*, *MAP3K13*, *PBRM1*, *SALL4*, *TBX3*	*CDH1*, *CDKN1B*, ***ERBB2***, *KEAP1*	***BARD1***, *CASP8*, *CCND1*, ***ESR1***, *ETV6*, *MAP2K4*, *NOTCH1*, ***NTRK3***, ***PIK3CA***, *RB1*, ***TP53***	***AKT1***, ***BARD1***, *CDH1*, *CDKN1B*, ***ESR1***, *FOXA1*, *GATA3*, *IRS4*, *PBRM1*, *RB1*	*CCND1*, *CTCF*, *MAP3K13*, *NOTCH1*, *TBX3*, ***TP53***	*ARID1B*, *BAP1*, *MAP3K1*, ***NTRK3***, ***PIK3CA***
NSCLC	*CCDC6*, *DROSHA*, *ERBB4*, *LRIG3*, ***MAP2K1***, *PTPN13*, *SDC4*, *SMARCA4*	****ALK****, ***ERBB2***, *HIP1*	****BRAF****, *NFE2L2*, *NKX2–1*	****ALK****, *CCDC6*, *DDR2*, *DROSHA*, *EML4*, *ERBB4*, *HIP1*, ***RET***, ***ROS1***, *SDC4*, *SLC34A2*	*MAP2K2*, *NFE2L2*, ***NRG1***	* ***BRAF*** *
CRC	****AKT1****, *APC*, *AXIN1*, *AXIN2*, *BAX*, *BCL9L*, ****BRAF****, *CTNNB1*, *FBXW7*, ***MAP2K1***, ****MLH1****, ****NTRK2****, ****PIK3CA****, *PIK3R1*, *PTPRT*, *QKI*, *RSPO2*, *SMAD3*, *SMAD4*, *TBL1XR1*, *UBR5*	*B2M*, *GRIN2A*, *RSPO3*, *TCF7L2*	*EIF3E*, ***TP53***	****AKT1****, *AXIN1*, *AXIN2*, *B2M*, *CTNNB1*, *IKZF3*, ***MDM2***, ****MLH1****, ****NTRK2****, *RAD21*, *RSPO3*, *SFRP4*, *SMAD4*, *SRC*, *TBL1XR1*	****BRAF****, *EIF3E*, *FBXW7*, *MSH2*, ****PIK3CA****	
PRAD	*FOXA1*	*SALL4*, *TMPRSS2*		*ACSL3*, ***BRAF***, *CANT1*, *ELK4*, *ETV4*, *FOXA1*, *KLF6*, *NCOR2*, *SALL4*, *SLC45A3*, *TMPRSS2*, *ZFHX3*	*HERPUD1*	
SKCM	*B2M*, *BAP1*, *FAT4*, ***MAP2K1***, *PTPRT*, *SALL4*, *STAG2*, *TERT*	****CDKN2A****, *GNAQ*, *GRIN2A*, *MITF*, ****NRAS****	* ***BRAF*** *	*BAP1*, ****CDKN2A****, *ERBB4*, *GNAQ*, ***MAP2K2***, *MITF*, *RAC1*, *TERT*	* ***NRAS*** *	* ***BRAF*** *
CML	*BCR*, *ETNK1*, *GATA2*, *MECOM*, *MSI2*, ****PDGFRB****, *RPL22*, *SETBP1*	*CCDC6*		*BCR*, *CSF3R*, *ETNK1*, ***JAK2***, *MSI2*, ****PDGFRB****, *RPL22*	*CCDC6*, *SETBP1*	
GB			*DAXX*, ***PDGFRA***, *SALL4*			

**Fig 5:**
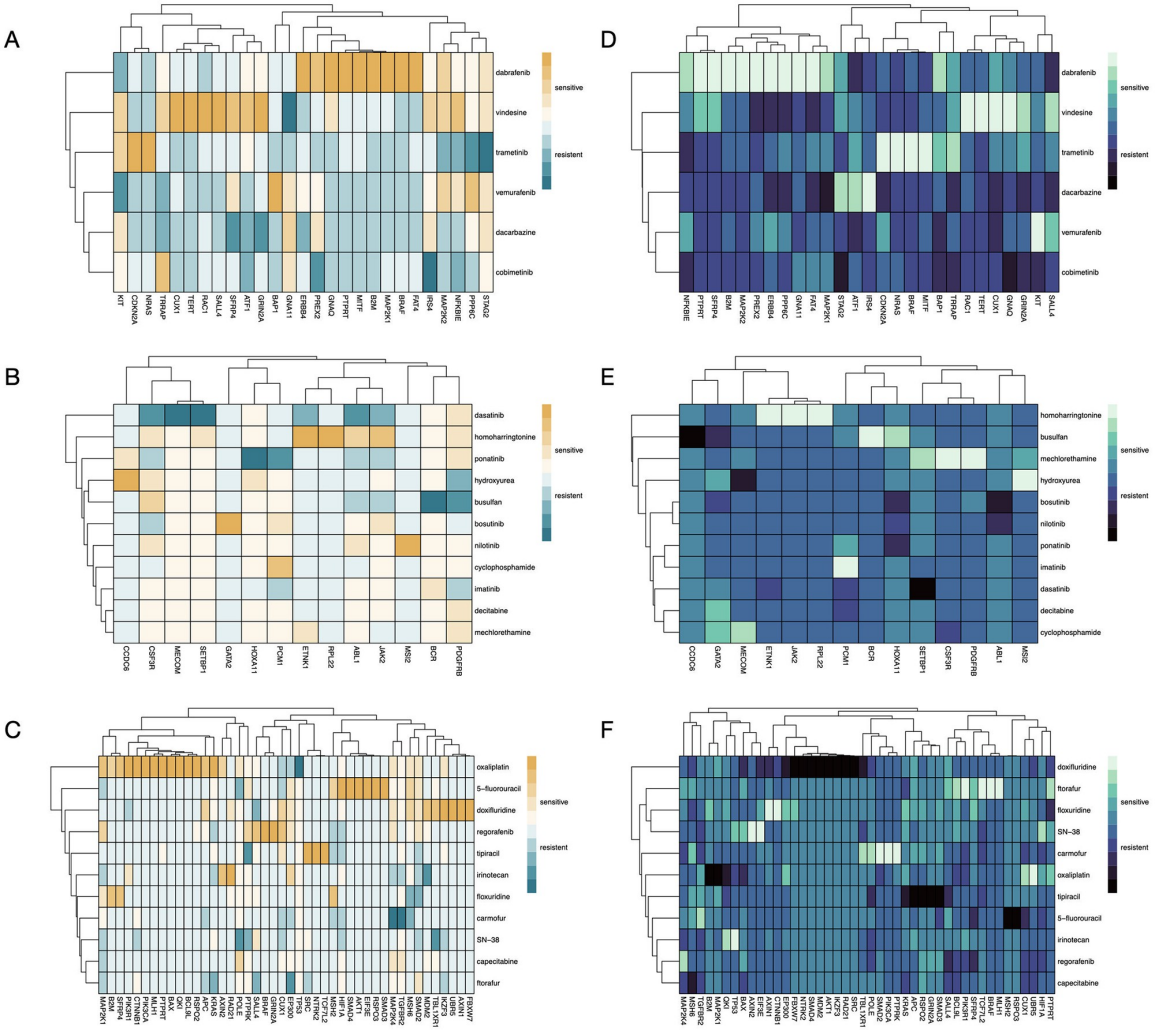
Gene-drug sensitivity maps for drug recommendation. A: Gene-drug AUC map for SKCM. B: Gene-drug AUC map for CML. C: Gene-drug AUC map for CRC. D: Gene-drug EC50 map for SKCM. E: Gene-drug EC50 map for CML. F: Gene-drug EC50 map for CRC.

Furthermore, based on the p-values of t-tests, we construct a gene-AUC map and a gene-EC50 map for BRCA, NSCLC, CRC, PRAD, and CML. The results are as shown in Fig 5 and S10 Fig. In each map, the rows represent genes, and the columns represent drugs. According to the effect of gene mutation on drug response, the color of each cell represents the degree of sensitivity or resistance, which is determined by the reciprocal of the p-value. The maps are able to provide drug recommendations given the genotypes of patients. To clarify, we take Fig 5A as an example, which shows the gene-AUC map of SKCM. The genotype of each patient can be divided into three types, with a single driver gene mutation, with multiple driver gene mutations, and with no driver gene mutation. In the first case, assuming that the gene with SNV is *GRIN2A*, then we should select the most sensitive drug in the column corresponding to *GRIN2A*, i.e. vindesine. In this way, a higher AUC is supposed to be achieved compared to the case where *GRIN2A* is not mutated. In the second case, multiple columns need to be combined for choosing the optimal drug. In the third case, where our gene-drug sensitivity map is not available, it can be observed from the Fig 3A that cobimetinib, dabrafenib, and trametinib are suitable options since the AUCs of the unmutated group are overall higher. For EC50, the goal is to select the drug with the lowest value. By comparing Fig 5A and 5D, it shows that similarity exists as for the effects of genetic mutations on drug AUC and EC50.

### High driver CNVs lead to variations in drug response

**Fig 6:**
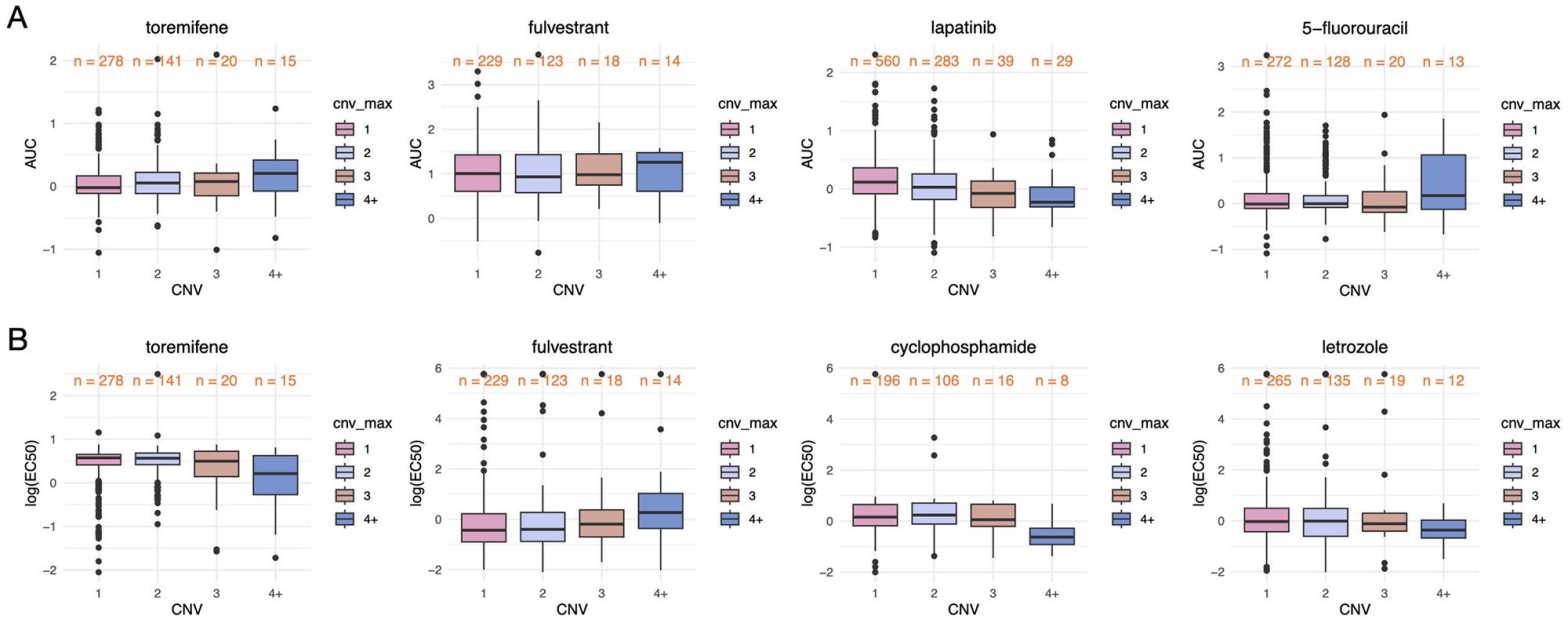
Box plots of the correlation between the maximum CNVs in driver genes and BRCA drug responses. A: AUC box plots of 4 BRCA drugs. B: EC50 box plots of 4 BRCA drugs.

For gene CNVs, we first evaluate the impact of the max value among driver genes on drug response, where the real-valued CNVs are adjusted into integers. Fig 6, S11 and S12 Figs show the results of a list of BRCA and CML drugs, respectively. It can be seen from the box and line plots that an increasing or decreasing trend exists between CNV and drug response across multiple drugs. Especially, when the maximum CNV is greater than three, the drug response shows significant differences. Such finding confirms the conclusion of [16] which studied 21 HGSOC samples. We show that this conclusion can be extended to several other cancers. In addition, it can also be seen that for some drugs such as toremifene, bosutinib, and hydroxyurea, the AUC and EC50 show opposite trends as CNV increases. Such result is reasonable given that AUC and EC50 are opposite to some extent in measuring drug response. However, some other drugs such as fulvestrant do not show the same pattern. This is believed to be caused by the lack of strong correlation between the two measures and the low number of samples with high CNV.

To further examine the relationship between CNV and drug response more specifically, we then focus on each driver gene. By calculating the three correlation coefficients, which are P.cor, S.rho, and K.tau, we find that the overall correlation between driver CNV and drug response is weak. S13 Fig shows the scatter plots of the relationship between the response of several BRCA drugs and the CNV of *IKZF3*, *ERBB2*, *BRCA2*, *AKT1*, and *ESR1*. Among the three correlation coefficients, the absolute value of P.cor and S.rho are around 0.18 to 0.2, which are higher than K.tau. In addition, the three fitting models, which are LM, RLM and GAM, also fail to capture the significant linear or non-linear relationships between CNV and drug response. To sum up, although high driver CNVs lead to significant differences in drug response, the overall correlation between CNV and drug response is relatively low. Based on the Pearson correlation coefficients between driver CNVs and drug response, we also construct a series of gene-AUC and gene-EC50 maps, which are shown in S14 Fig.

### Strong correlation exists between driver CNVs and drug response in cancer-specific samples

**Fig 7:**
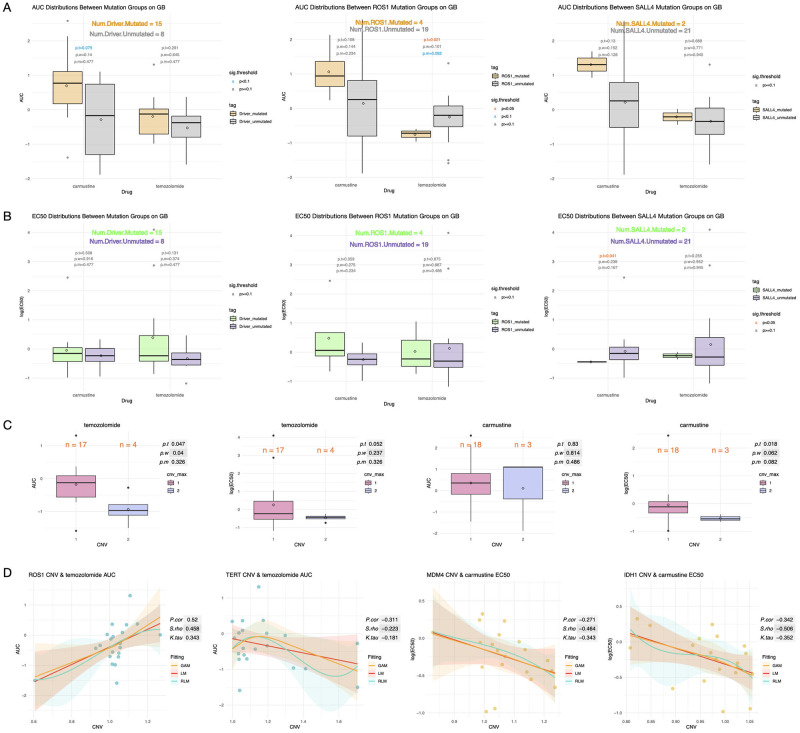
Summary of drug response analysis on GB samples. A: Box plots showing AUC distributions of GB drugs with or without SNVs of GB driver genes. B: Box plots showing EC50 distributions of GB drugs with or without mutations of GB driver genes. C: Box plots showing the correlation between the maximum CNV in driver genes and GB drug response. D: Scatter plots showing the correlation of CNV and GB drug response.

We segment the samples by cancer type and further examine the relationship between genetic mutations and drug response. The results of GB are summarized in the Fig 7. In this case, the total number of samples is 23. In Fig 7A and 7B, although significant differences between groups can be observed in the box plots, the statistical test results do not show strong significances due to lack of samples. Among the 14 GB driver genes, we find that *ROS1* shows significance for the AUC of temozolomide by t-test and median test, while *SALL4* shows significance for the EC50 of carmustine by t-test. In addition, by rounding up the CNV values, the samples can be divided into two groups. According to Fig 7C, the AUC of temozolomide and the EC50 of carmustine show significant differences between CNV groups. Moreover, we find that compared to the analysis of pan-cancer CNVs, it can be seen from Fig 7D that there is a stronger correlation between drug response and the CNVs of genes such as *ROS1*, *TERT*, *MDM4*, and *IDH1*. Among these genes, *ROS1* and *IDH1* are included in OncoKB as predictive biomarkers for drug response, while *TERT* and *MDM4* also show the potential to predict drug response.

We also study 46 NSCLC samples and the results are summarized in S15 Fig, where same conclusions as the GB analysis can be reached. According to S15D Fig, a group of genes *HIP1*, *CD74*, *LRIG3*, and *KDR* are potential biomarkers for drug response which achieve drug response correlations close to known biomarkers including *EGFR*, *MAP2K2*, and *ALK*.

### Analysis of potential predictors for drug LFC

**Fig 8:**
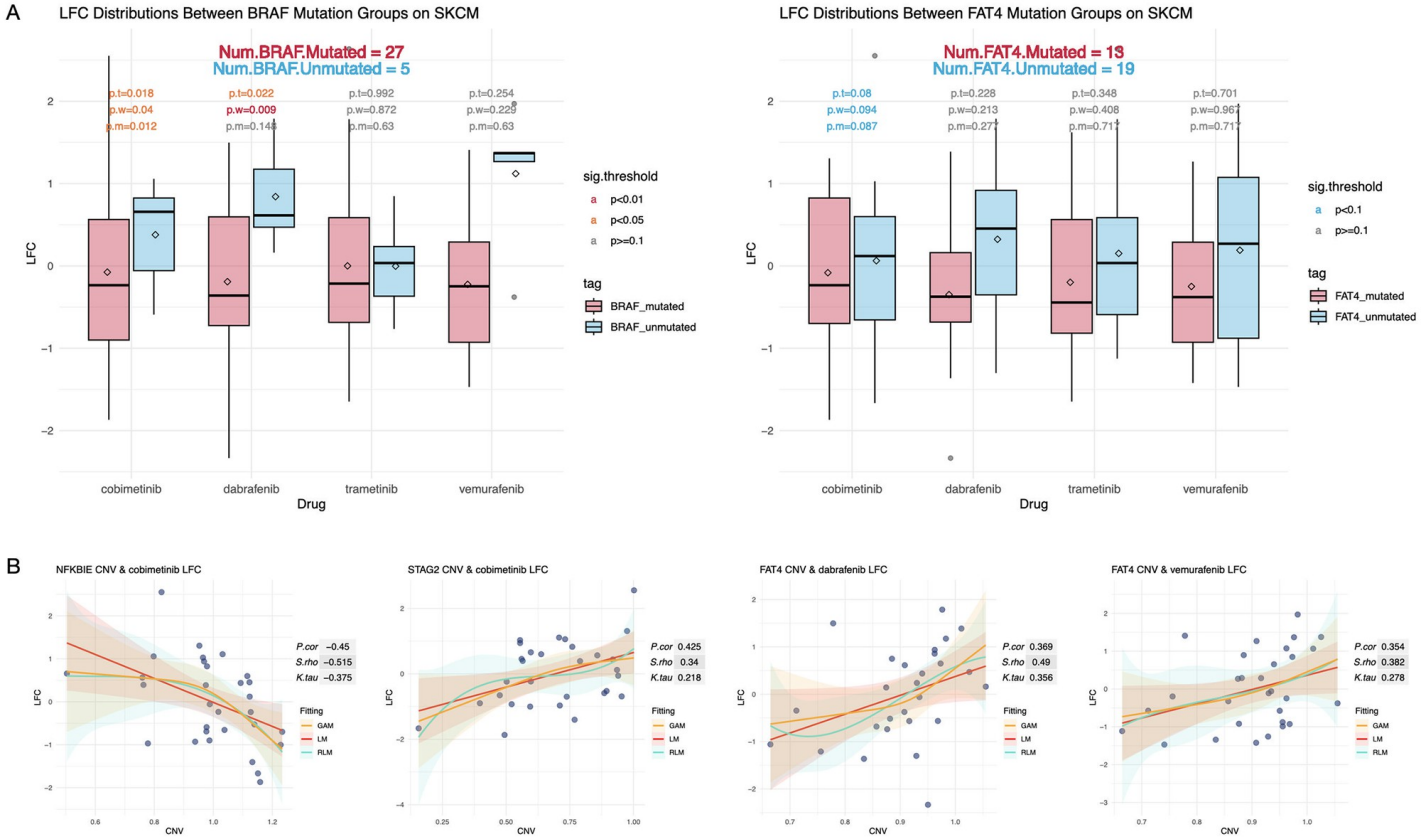
Summary of LFC analysis on SKCM drugs. A: Box plots showing LFC distributions of SKCM drugs with or without SNVs of *BRAF* and *FAT4*. B: Scatter plots of drug LFCs and the CNVs of *NFKBIE*, *STAG2*, and *FAT4*.

Finally, we study the potential relationship between LFC and driver gene mutations. Experiments are conducted on three types of cancers, namely SKCM, CRC, and NSCLC. The results of SKCM are summarized in the Fig 8, S16 and S17 Figs. Results show that similar to AUC and EC50, LFC can also be affected by driver gene mutations. According to Fig 8A, the significance of *BRAF* can be verified for the LFC of cobimetinib and dabrafenib, which is consistent with AUC and EC50 results. We also find that the CNVs of driver genes including *NFKBIE*, *STAG2*, and *FAT4* are predictive of drug LFC based on their correlations. According to S16 and S17 Figs, a group of genes including *PTPRT*, *BAX*, *SALL4*, *SRC*, *EZR*, *RBM10*, *CD74*, and *IQCJ.SCHIP1* also reveal the potential of predicting drug response based on the statistical results.

### Verification of discovered biomarkers on GDSC2

To increase the credibility of the results, we implement an experiment on the GDSC2 dataset. According to the distributions of drug AUC and IC50 shown in S18 Fig, the distribution of AUC lacks normality and is close to 1, while the distribution of IC50 has stronger normality. Therefore, IC50 is used for the experiment. For each driver gene and each drug, we analyze the difference between the two populations with or without SNVs based on three statistical tests. The resulting genes of BRCA are summarized in Table 3. By applying the screening criteria that three p-values are less than 0.05 or at least one p-value is less than 0.01, two genes *ERBB2* and *PIK3CA* are identified as drug response biomarkers. When the criteria is relaxed to at least two p-values are less than 0.05, the resulting genes are *FOXA1*, *BAP1*, *NTRK3*, *ERBB2*, *ETV6*, and *PIK3CA*. It can be seen from Table 2 that all these genes are included in the CCLE results. We also apply the same methods on PRAD and find two significant genes *NCOR2* and *ERG*, where *NCOR2* is also found based on the CCLE dataset. The analyses related to drug AUC and gene CNV are not fully achievable considering the corresponding distribution differences of the CCLE and GDSC2. In general, although the drug response biomarker identification results in GDSC are not identical to the CCLE results due to differences in datasets and measurements, the GDSC results provide support for a series of discovered biomarkers.

**Table 3:** Driver genes that can serve as IC50 biomarkers by SNV for BRCA drugs based on GDSC2 dataset. P-values less than 0.05 are underlined. Genes in bold represent confirmed drug response biomarkers included in OncoKB.

Gene	Drug	p.t	p.w	p.m	No. mutated	No. unmutated
*FOXA1*	paclitaxel	0.011	0.037	0.157	2	937
*BAP1*	paclitaxel	0.011	0.014	0.068	15	924
*NTRK3*	tamoxifen	0.025	0.055	0.014	6	932
***ERBB2***	gemcitabine	0.031	0.032	0.019	15	927
*ETV6*	tamoxifen	0.053	0.034	0.033	8	930
***PIK3CA***	5-fluorouracil	0.085	0.049	0.009	94	855

## Discussion

The high heterogeneity of cancer results in differences in drug response. Previous analyses identified putative cancer drivers but their impacts on drug response have not been fully clarified. In this study, we test whether SNVs and CNVs of driver genes affect the response of cancer drugs. By studying a variety of cancer drugs in the CCLE database, the results suggest that mutations in driver genes can significantly affect the response to specific drugs. Overall, we find that the impacts of SNVs on drug AUC are more significant than that on EC50. Treating driver genes as a set or individually, their mutations have a consistent pattern of impact on drug response. We validate the ability of drug response prediction for several known biomarkers including *BRAF*, *NRAS*, *MAP2K1*, *MAP2K2*, and *CDKN2A*. Moreover, by using the same evaluation criteria, we identify new biomarkers whose impact on drug response spans multiple cancers, including *SALL4*, *B2M*, *BAP1*, *CCDC6*, *ERBB4*, *FOXA1*, *GRIN2A*, and *PTPRT*.

The drug sensitivity matrices shown in Fig 5 and S10 Fig can be used to provide medication recommendations based on the genetic mutation status of patients. The driver genes and drugs are clustered in the matrices, from which the relationships among drugs and genes can be inferred. It can be seen from Fig 5A that 2 SKCM drugs, namely dabrafenib and vindesine show nearly opposite behavior according to gene SNVs. Similar behavior exists in 2 drugs for CML, namely dasatinib and homoharringtonine, according to Fig 5B. Such property can provide valuable references for drug recommendation.

Our study on the CNVs of driver genes shows that a high CNV level can influence certain drug responses. For pan-cancer samples, the correlations between drug responses and CNVs are low in general. However, when screening samples with specific cancers, a stronger linear relationship between CNV and drug response is demonstrated. In order to eliminate accidental factors, we validate on multiple cancer samples. However, the number of cancer-specific samples in the CCLE database is still limited. Thus, more solid proofs rely on future works.

By focusing on the driver genes, the sparse problem of mutation matrix can be effectively avoid, which increases the credibility of results. In this study, we have shown that a bunch of driver genes could be clinical biomarkers for drug response prediction. However, although the discovered biomarkers show significance between mutation groups, we find that the magnitude of the differences between the groups are not huge. Therefore, the predicting performance based on the genes alone are not good enough at present. We believe that future studies will benefit from our discovery and promote the robustness of predicting models. In addition, with low-depth whole-genome sequencing becoming increasingly affordable and available, genetic-based models will be greatly improved in practice.

## Conclusion

In pursuit of precision medicine for cancer, we study the relationship between driver gene mutations and drug response based on the CCLE database. By analyzing gene CNVs and SNVs respectively, we confirm our proposed hypothesis from multiple perspectives. Results show that mutations in certain driver genes not only contribute to tumor progression, but also significantly affect the response to anti-cancer drugs. The findings may provide important clues to further understand drug response and cancer treatment.

## Supporting information

S1 Fig Correlation of AUC and log(EC50) on cancer drugs. A: BRCA. B: NSCLC. C: CRC. D: PRAD. E: SKCM. F: CML. G: GB.
(TIFF)

S2 Fig Distribution of driver gene CNV. A: Empirical and theoretical density of CNV. B: Empirical and theoretical cumulative distribution functions of CNV.
(TIFF)

S3 Fig Distribution differences of drug responses between mutation groups. A: Box plots showing EC50 distributions of SKCM drugs with or without SNVs of SKCM driver genes. B: Box plots showing EC50 distributions of NSCLC drugs with or without SNVs of NSCLC driver genes. The diamond shapes represent mean values.
(TIFF)

S4 Fig Correlations of the significance ranks of SKCM drugs by three statistical tests. A: Correlation of the significance between t-test and Wilcoxon-test on AUC. B: Correlation of the significance between t-test and median-test on AUC. C: Correlation of the significance between Wilcoxon-test and median-test on AUC. D: Correlation of the significance between t-test and Wilcoxon-test on AUC. E: Correlation of the significance between t-test and median-test on AUC. F: Correlation of the significance between Wilcoxon-test and median-test on AUC. G: Correlation of the significance between AUC and EC50 by t-test. H: Correlation of the significance between AUC and EC50 by Wilcoxon-test. I: Correlation of the significance between AUC and EC50 by median-test.
(TIFF)

S5 Fig Correlations of the significance ranks of NSCLC drugs by three statistical tests. Subtitles are identical to S4 Fig.
(TIFF)

S6 Fig Summary of *NRAS* mutations affecting SKCM drug responses. (TIFF)

S7 Fig Summary of *GRIN2A* mutations affecting SKCM drug responses. (TIFF)

S8 Fig Summary of *CDKN2A* mutations affecting SKCM drug responses. (TIFF)

S9 Fig Summary of *FAT4* mutations affecting SKCM drug responses. (TIFF)

S10 Fig Gene-drug sensitivity maps for drug recommendation. A: Gene-drug AUC map for NSCLC. B: Gene-drug AUC map for PRAD. C: Gene-drug AUC map for of BRCA. D: Gene-drug EC50 map for NSCLC. E: Gene-drug EC50 map for PRAD. F: Gene-drug EC50 map for BRCA.
(TIFF)

S11 Fig Line plots of the correlation between the maximum CNVs in driver genes and BRCA drug responses. A: AUC line plots of 4 BRCA drugs. B: EC50 line plots of 4 BRCA drugs.
(TIFF)

S12 Fig Box and line plots of the correlation between the maximum CNVs in driver genes and CML drug responses. A: AUC box and line plots of 4 CML drugs. B: EC50 box and line plots of 4 CML drugs.
(TIFF)

S13 Fig Plots showing the relations of driver gene CNVs and SKCM drug responses. (TIFF)

S14 Fig Gene-drug sensitivity maps of driver CNVs. A: Gene-drug AUC map for SKCM. B: Gene-drug AUC map for PRAD. C: Gene-drug AUC map for NSCLC. D: Gene-drug AUC map for CRC. E: Gene-drug AUC map for CML. F: Gene-drug AUC map for of BRCA. G: Gene-drug EC50 map for SKCM. H: Gene-drug EC50 map for PRAD. I: Gene-drug EC50 map for NSCLC. J: Gene-drug EC50 map for CRC. K: Gene-drug EC50 map for CML. L: Gene-drug EC50 map for BRCA.
(TIFF)

S15 Fig Summary of drug response analysis on NSCLC samples. A: Box plots showing AUC distributions of NSCLC drugs with or without SNVs of NSCLC driver genes. B: Box plots showing EC50 distributions of NSCLC drugs with or without mutations of NSCLC driver genes. C: Box plots showing the correlation between the maximum CNV in driver genes and NSCLC drug response. D: Scatter plots showing the correlation of CNV and NSCLC drug response.
(TIFF)

S16 Fig Summary of LFC analysis on CRC drugs. (TIFF)

S17 Fig Summary of LFC analysis on NSCLC drugs. (TIFF)

S18 Fig GDSC2 drug response distributions. A: Histogram of GDSC2 AUC. B: Histogram of GDSC2 IC50.
(TIFF)
