# Supplementary figures and images for: Single nucleotide and copy number variants of cancer driver genes inform drug response in multiple cancers
Source: PLoS One. 2024 Jul 31;19(7):e0306343. doi: 10.1371/journal.pone.0306343 (PMC11290640; doi:10.1371/journal.pone.0306343)

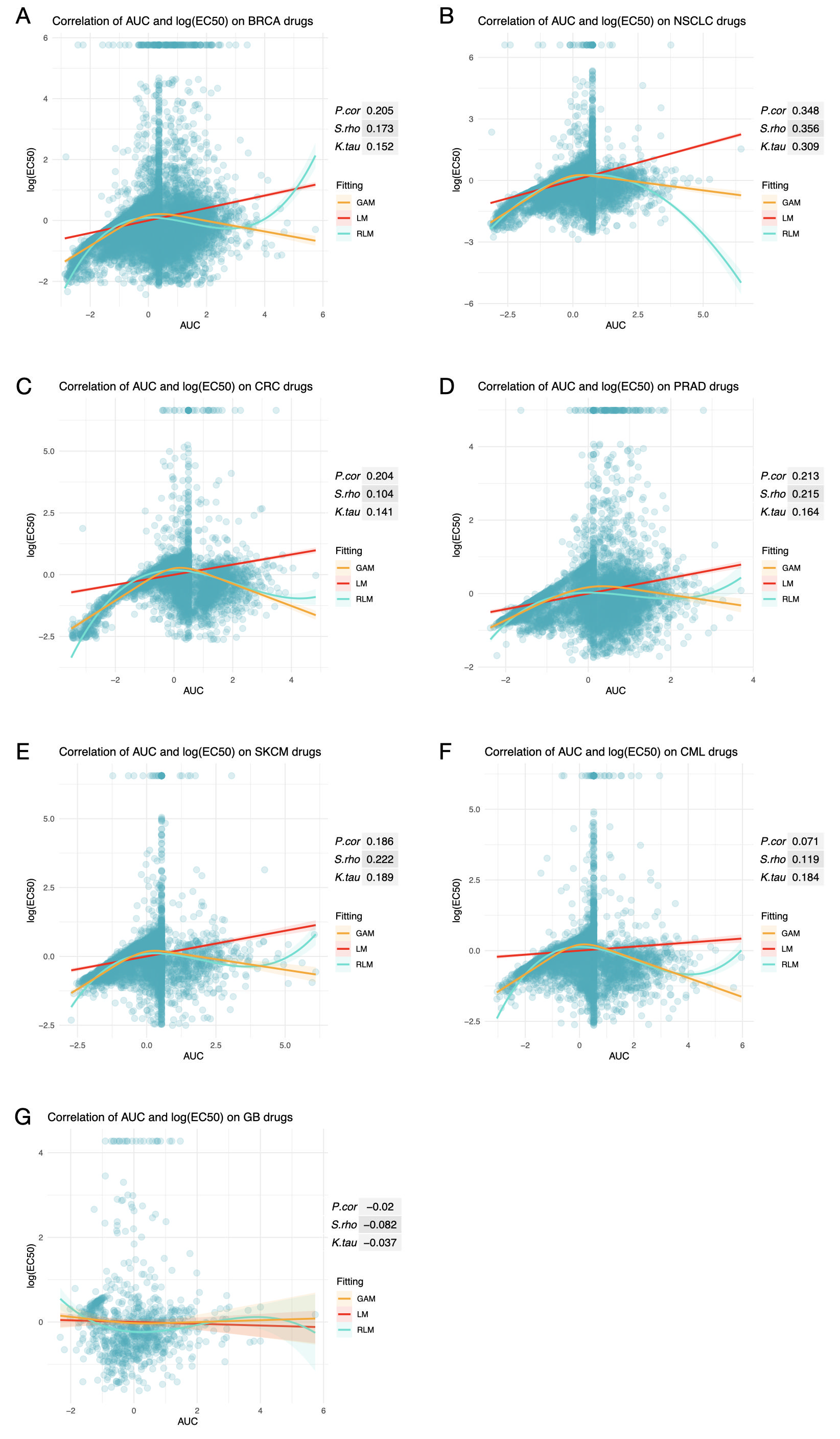

Supplement: S1 Fig — A: BRCA. B: NSCLC. C: CRC. D: PRAD. E: SKCM. F: CML. G: GB. (TIFF) [file pone.0306343.s001.tiff]

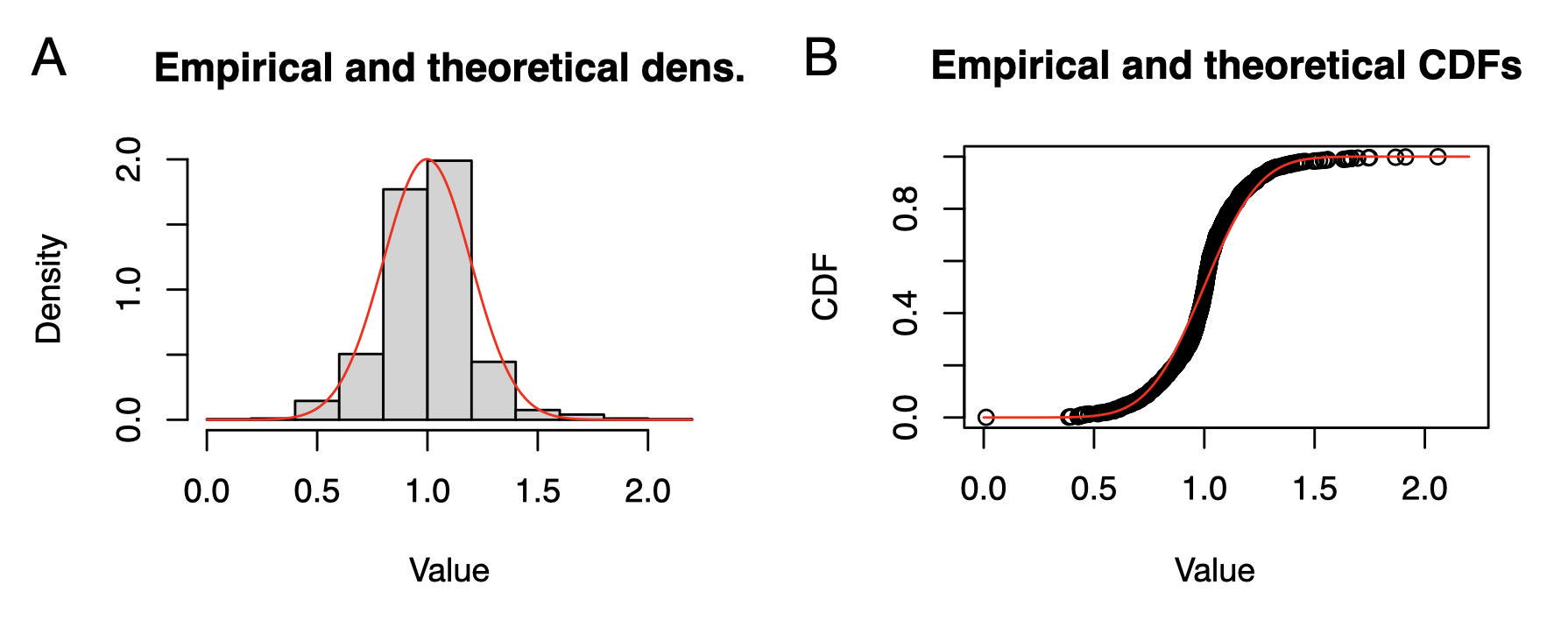

Supplement: S2 Fig — A: Empirical and theoretical density of CNV. B: Empirical and theoretical cumulative distribution functions of CNV. (TIFF) [file pone.0306343.s002.tiff]

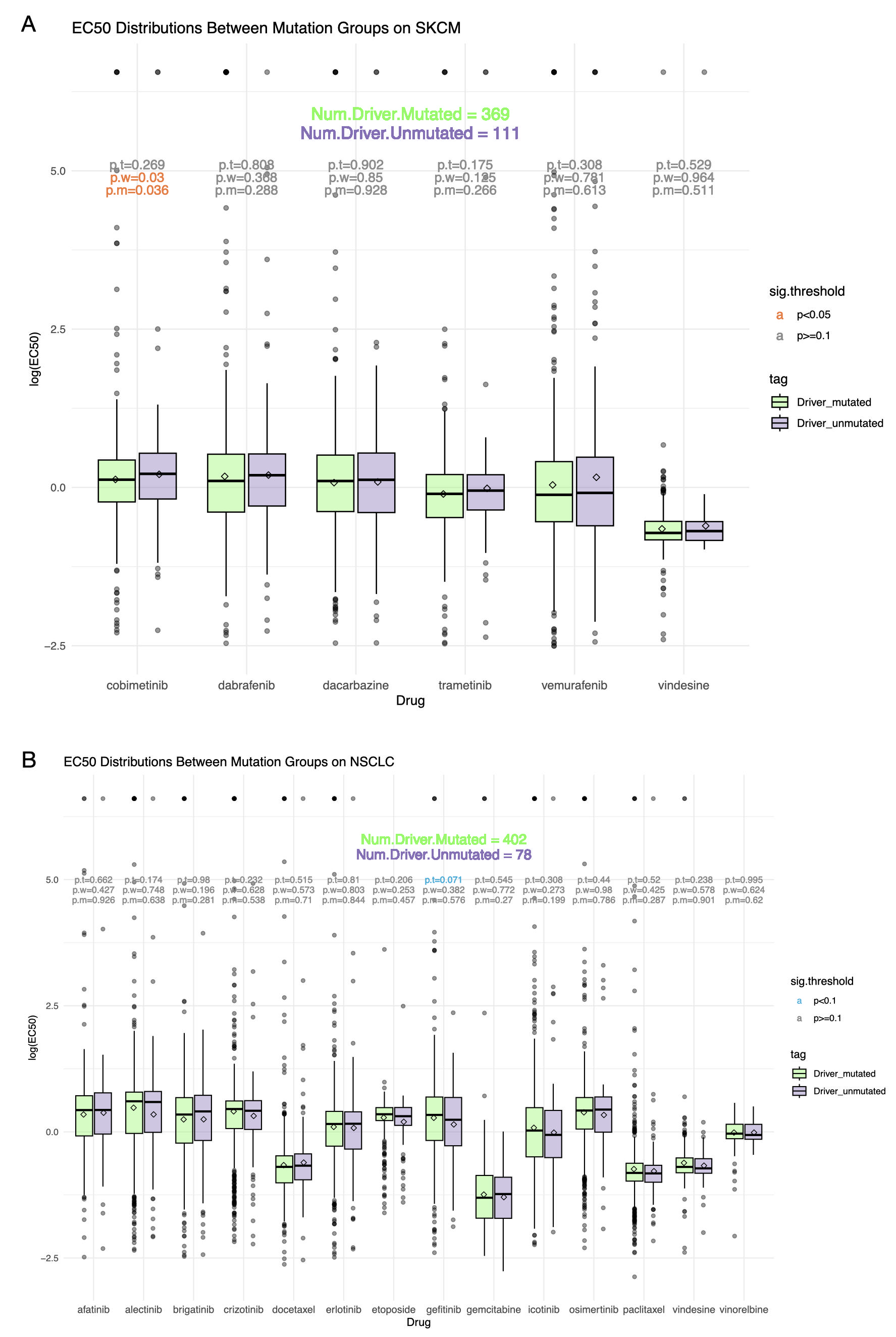

Supplement: S3 Fig — A: Box plots showing EC50 distributions of SKCM drugs with or without SNVs of SKCM driver genes. B: Box plots showing EC50 distributions of NSCLC drugs with or without SNVs of NSCLC driver genes. The diamond shapes represent mean values. (TIFF) [file pone.0306343.s003.tiff]

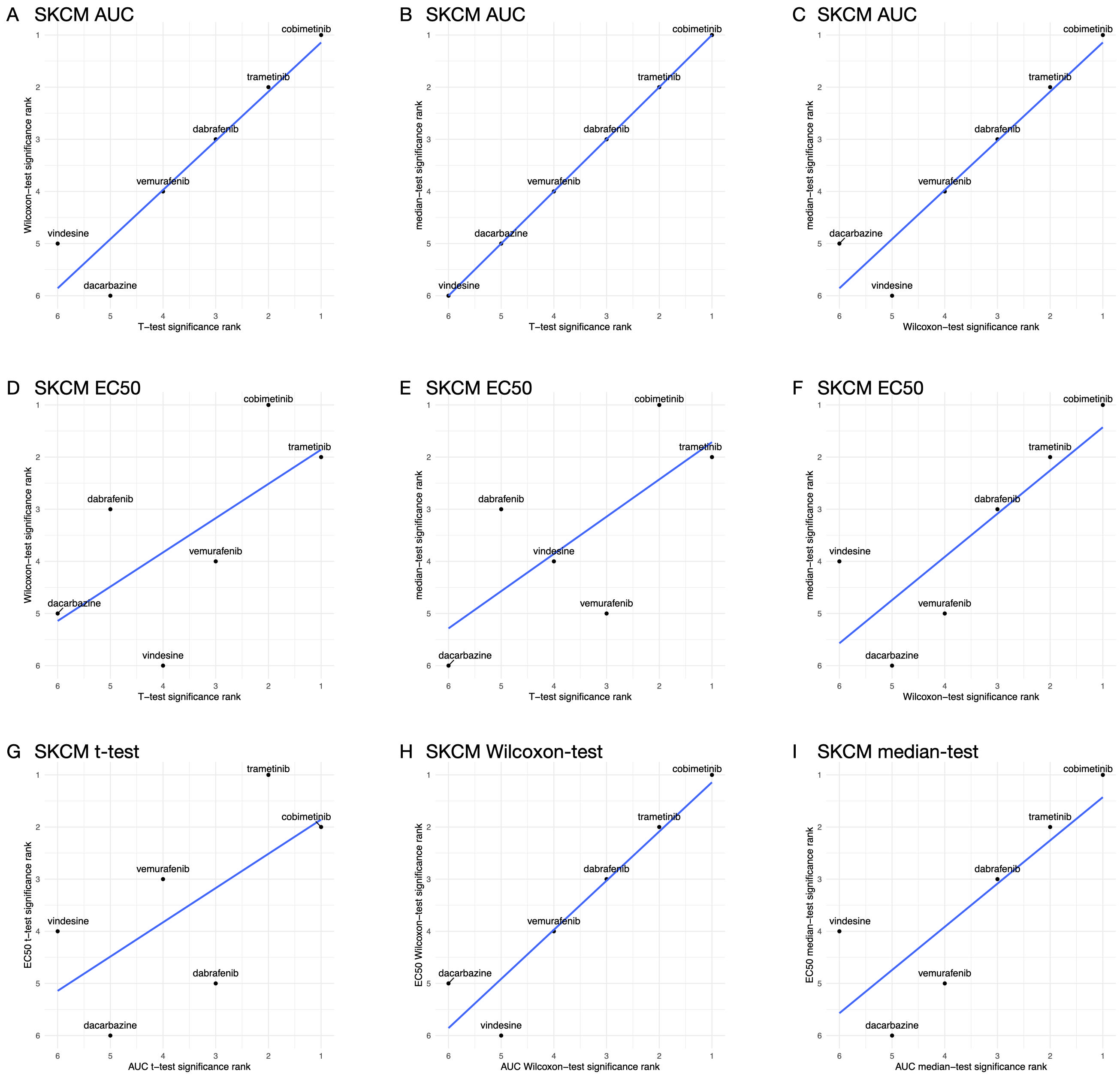

Supplement: S4 Fig — A: Correlation of the significance between t-test and Wilcoxon-test on AUC. B: Correlation of the significance between t-test and median-test on AUC. C: Correlation of the significance between Wilcoxon-test and median-test on AUC. D: Correlation of the significance between t-test and Wilcoxon-test on AUC. E: Correlation of the significance between t-test and median-test on AUC. F: Correlation of the significance between Wilcoxon-test and median-test on AUC. G: Correlation of the significance between AUC and EC50 by t-test. H: Correlation of the significance between AUC and EC50 by Wilcoxon-test. I: Correlation of the significance between AUC and EC50 by median-test. (TIFF) [file pone.0306343.s004.tiff]

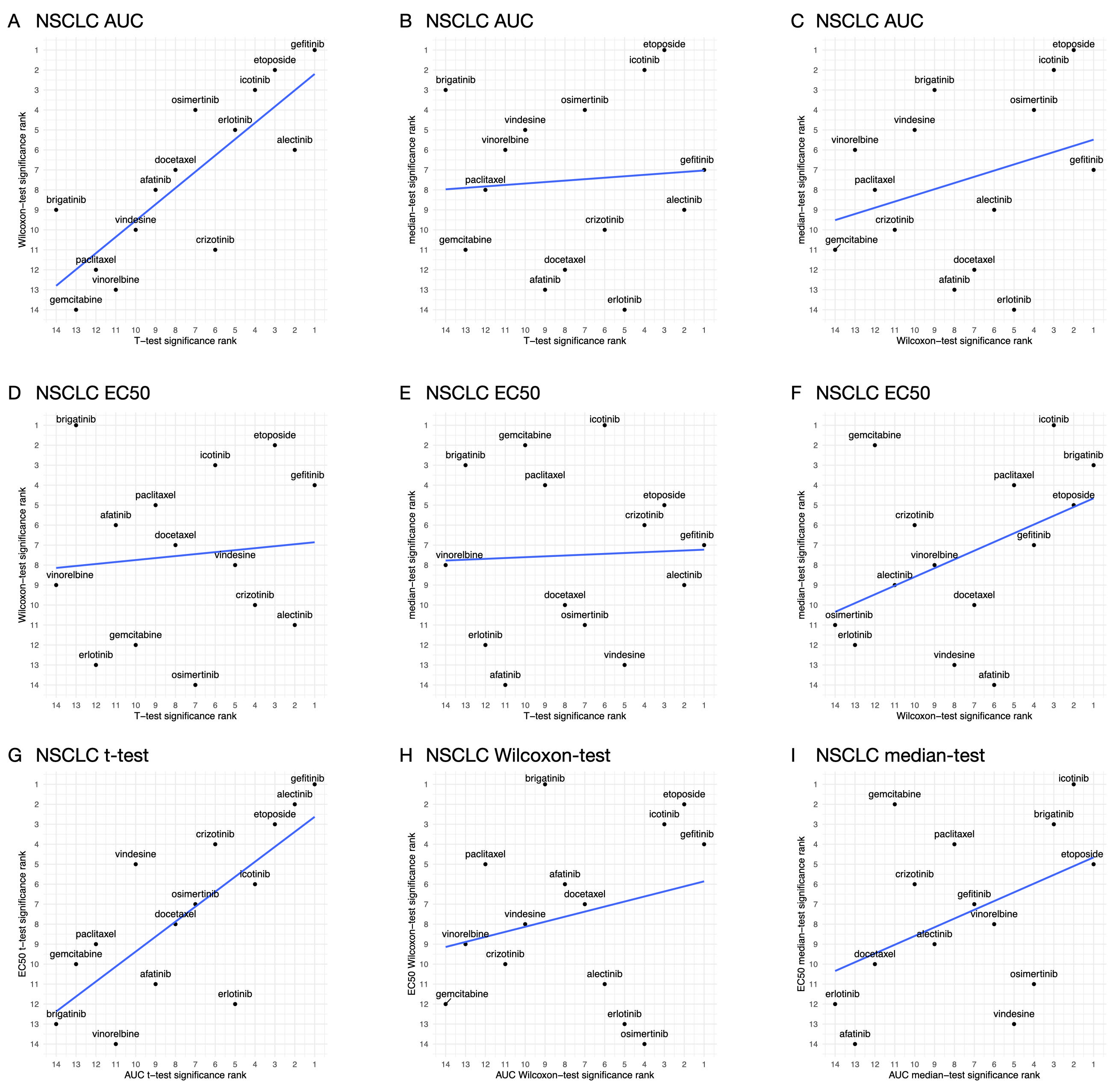

Supplement: S5 Fig — Subtitles are identical to S4 Fig. (TIFF) [file pone.0306343.s005.tiff]

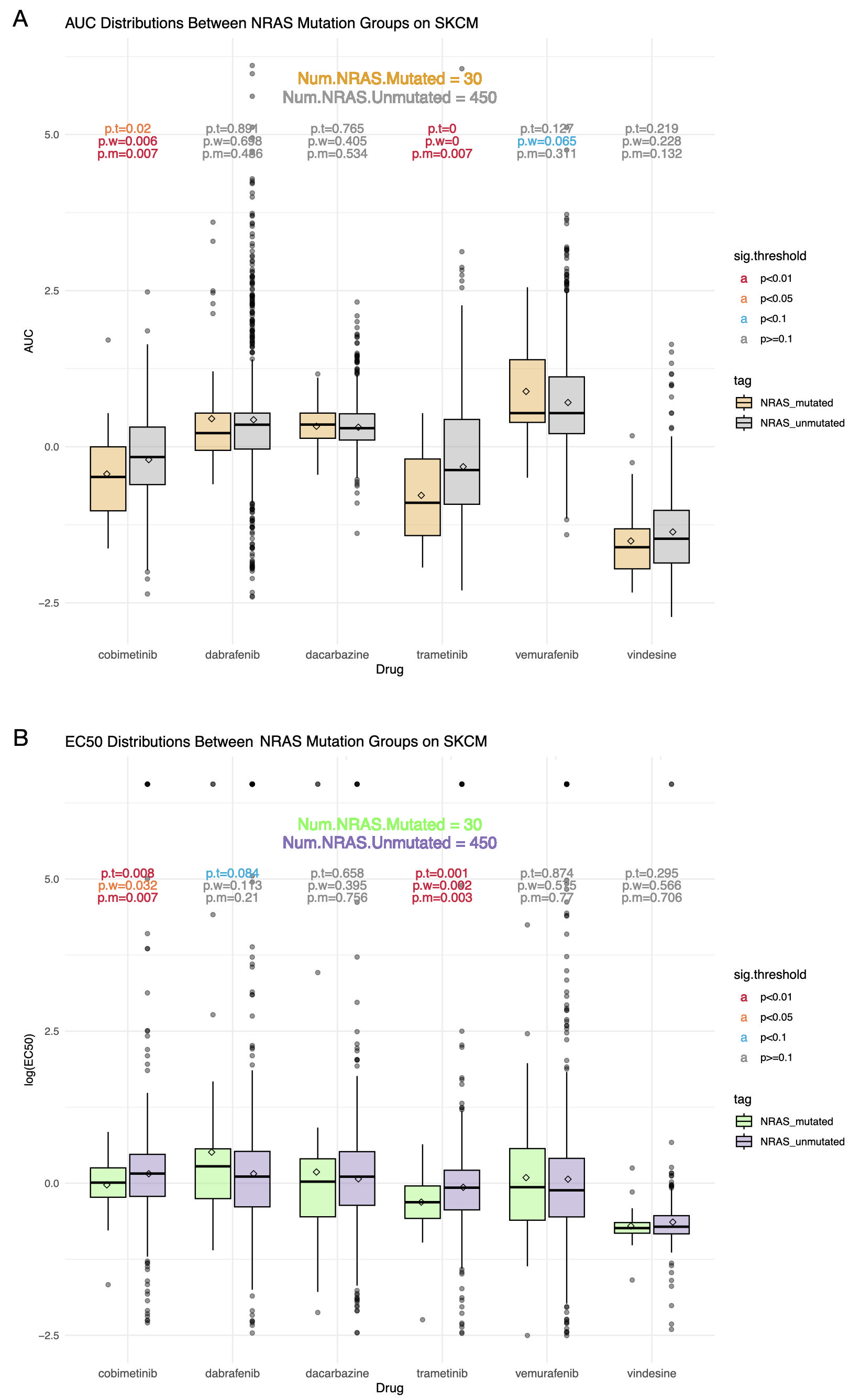

Supplement: S6 Fig — (TIFF) [file pone.0306343.s006.tiff]

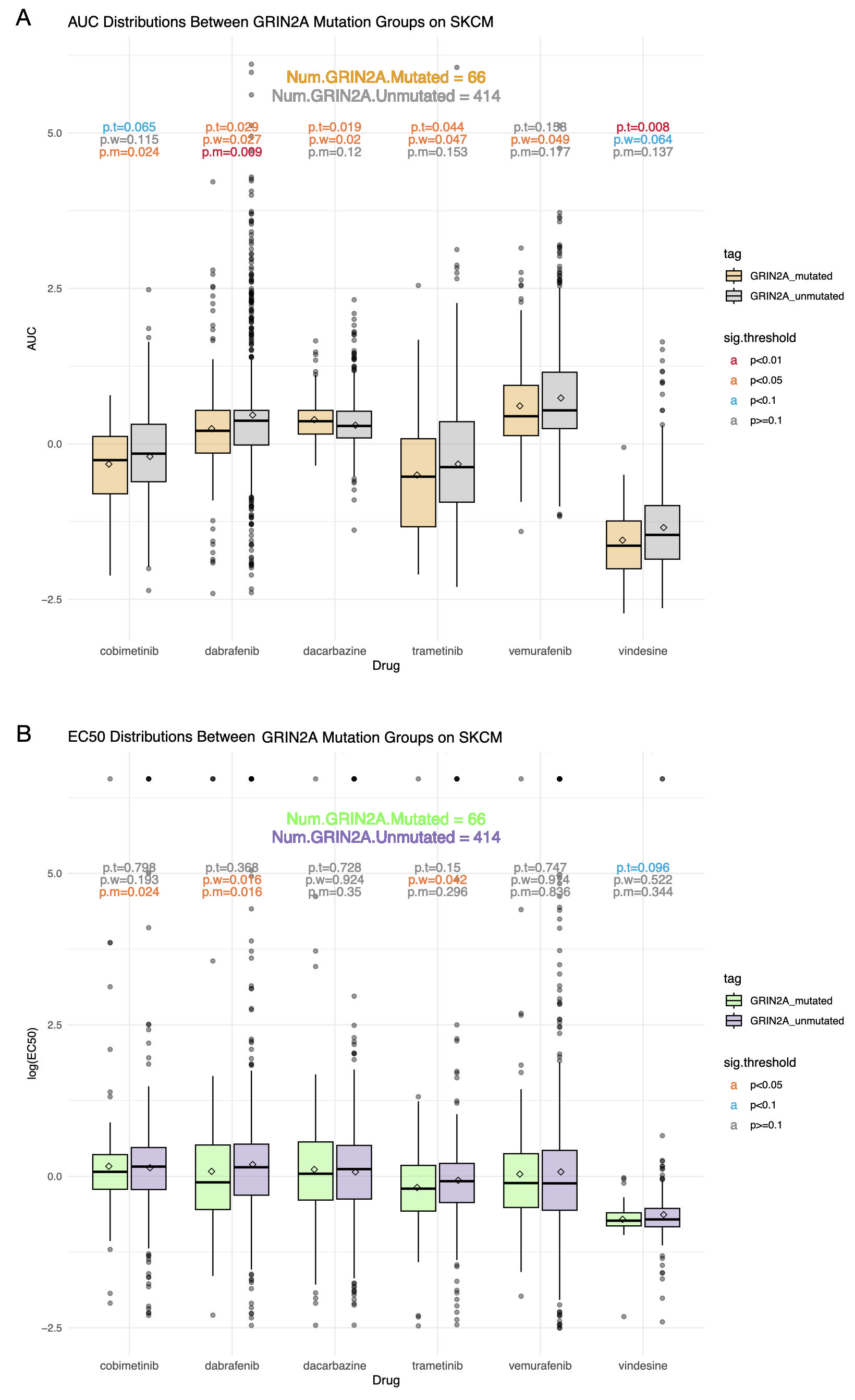

Supplement: S7 Fig — (TIFF) [file pone.0306343.s007.tiff]

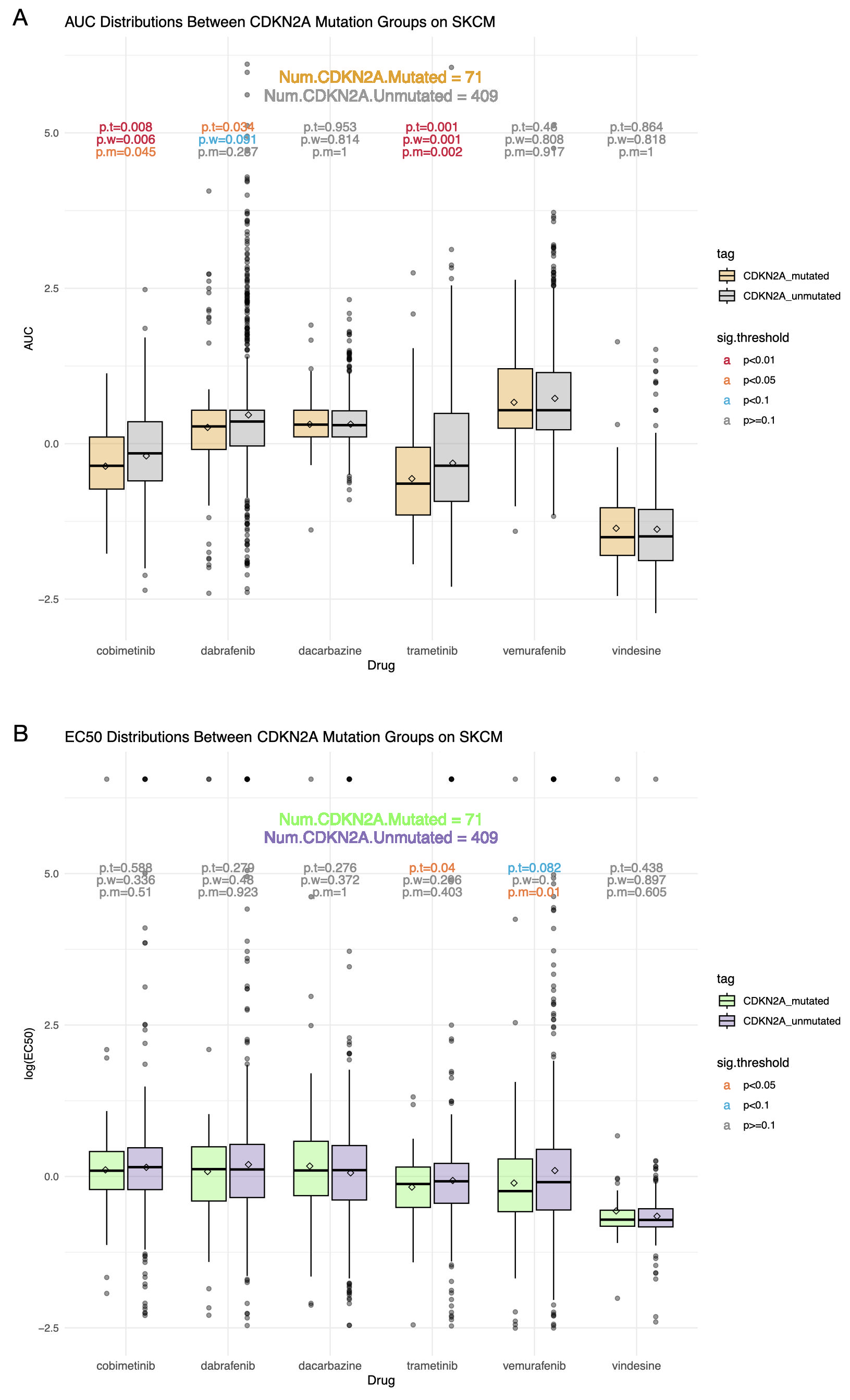

Supplement: S8 Fig — (TIFF) [file pone.0306343.s008.tiff]

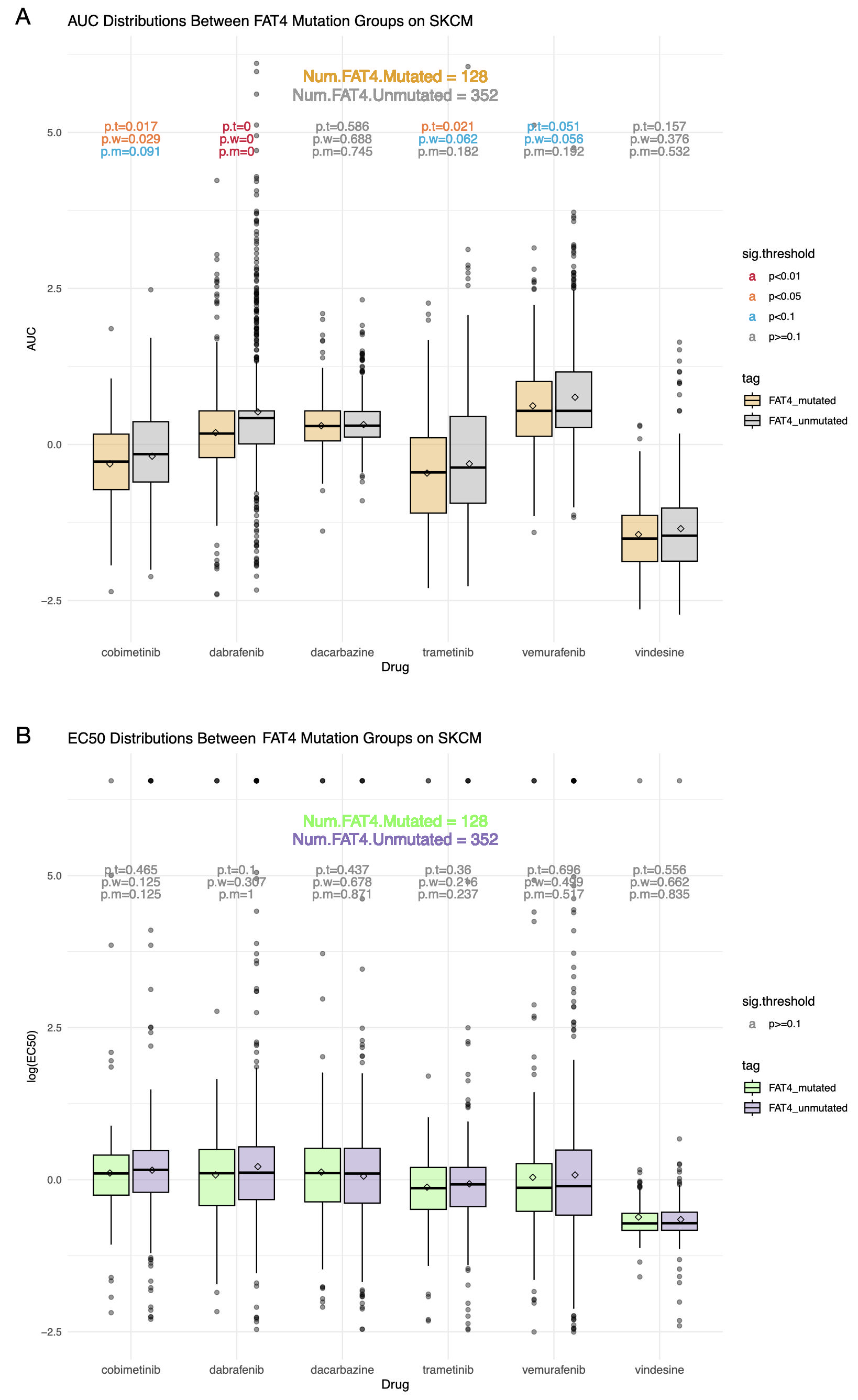

Supplement: S9 Fig — (TIFF) [file pone.0306343.s009.tiff]

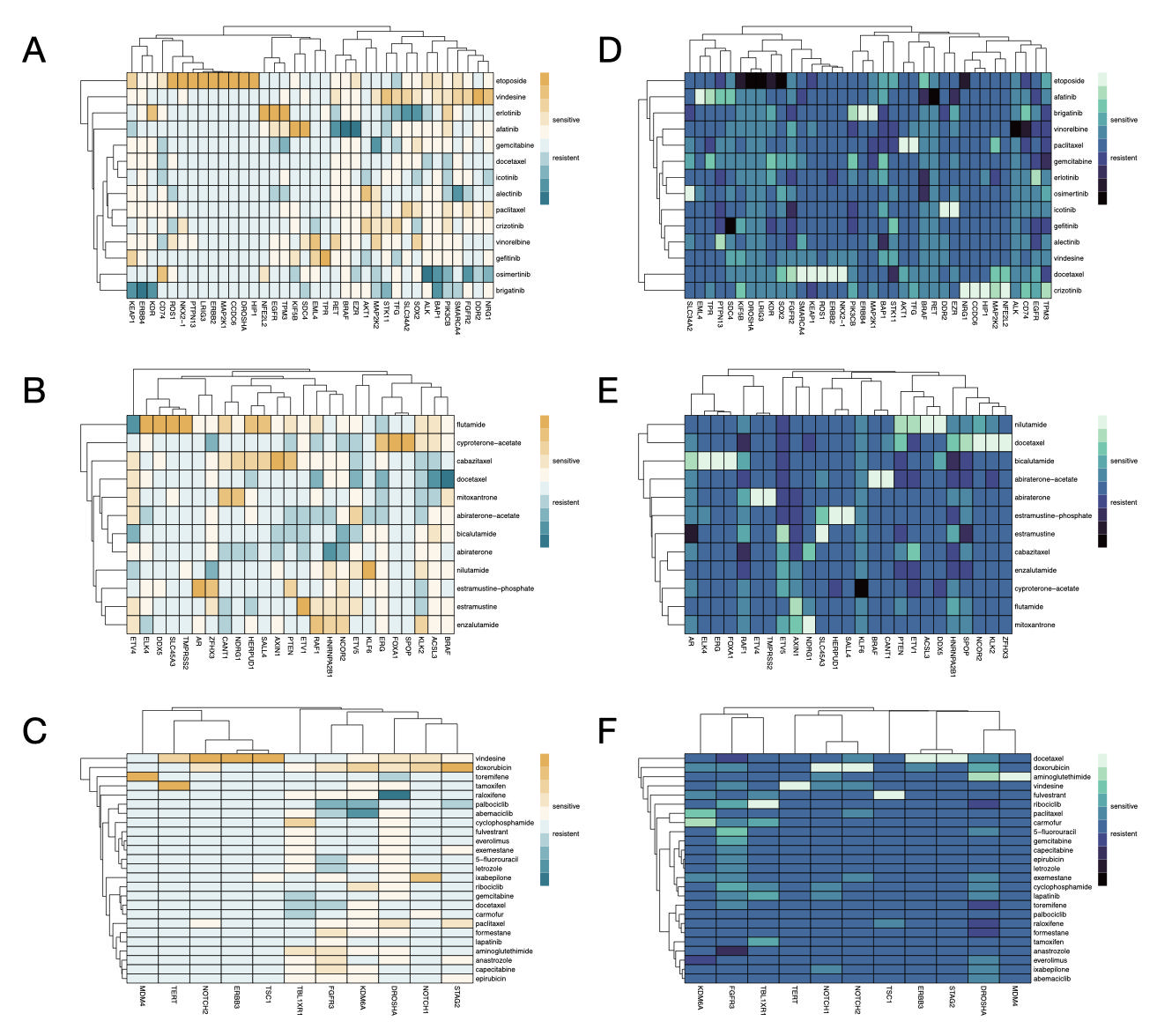

Supplement: S10 Fig — A: Gene-drug AUC map for NSCLC. B: Gene-drug AUC map for PRAD. C: Gene-drug AUC map for of BRCA. D: Gene-drug EC50 map for NSCLC. E: Gene-drug EC50 map for PRAD. F: Gene-drug EC50 map for BRCA. (TIFF) [file pone.0306343.s010.tiff]

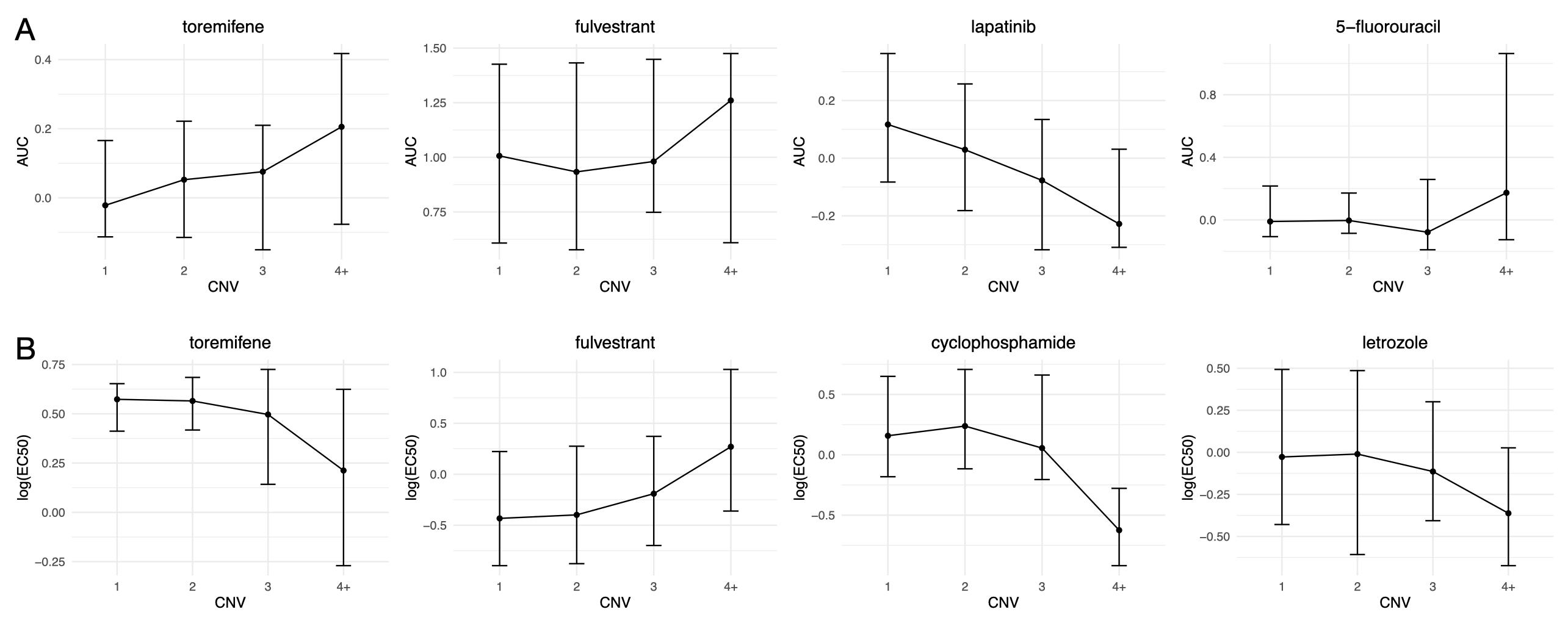

Supplement: S11 Fig — A: AUC line plots of 4 BRCA drugs. B: EC50 line plots of 4 BRCA drugs. (TIFF) [file pone.0306343.s011.tiff]

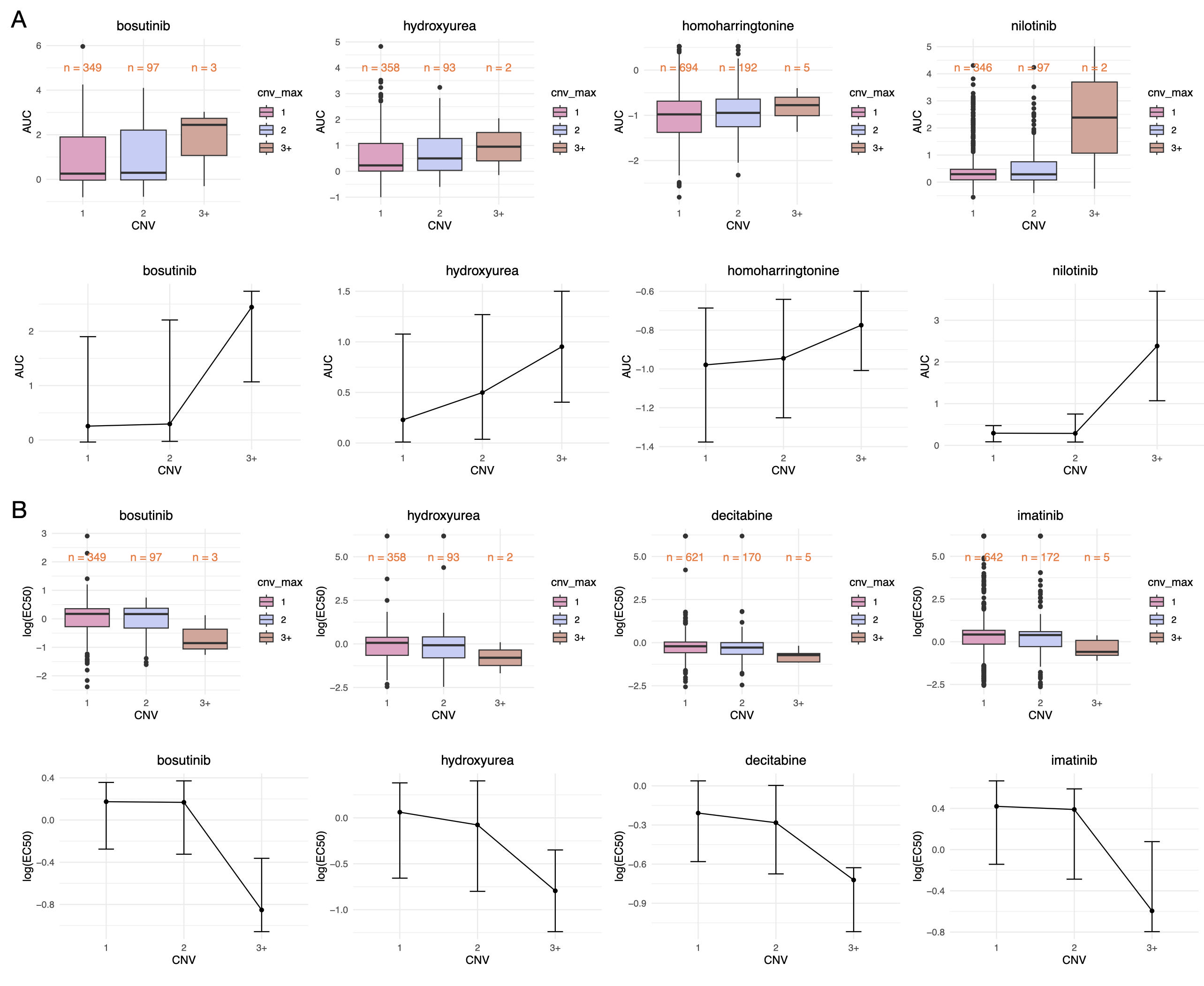

Supplement: S12 Fig — A: AUC box and line plots of 4 CML drugs. B: EC50 box and line plots of 4 CML drugs. (TIFF) [file pone.0306343.s012.tiff]

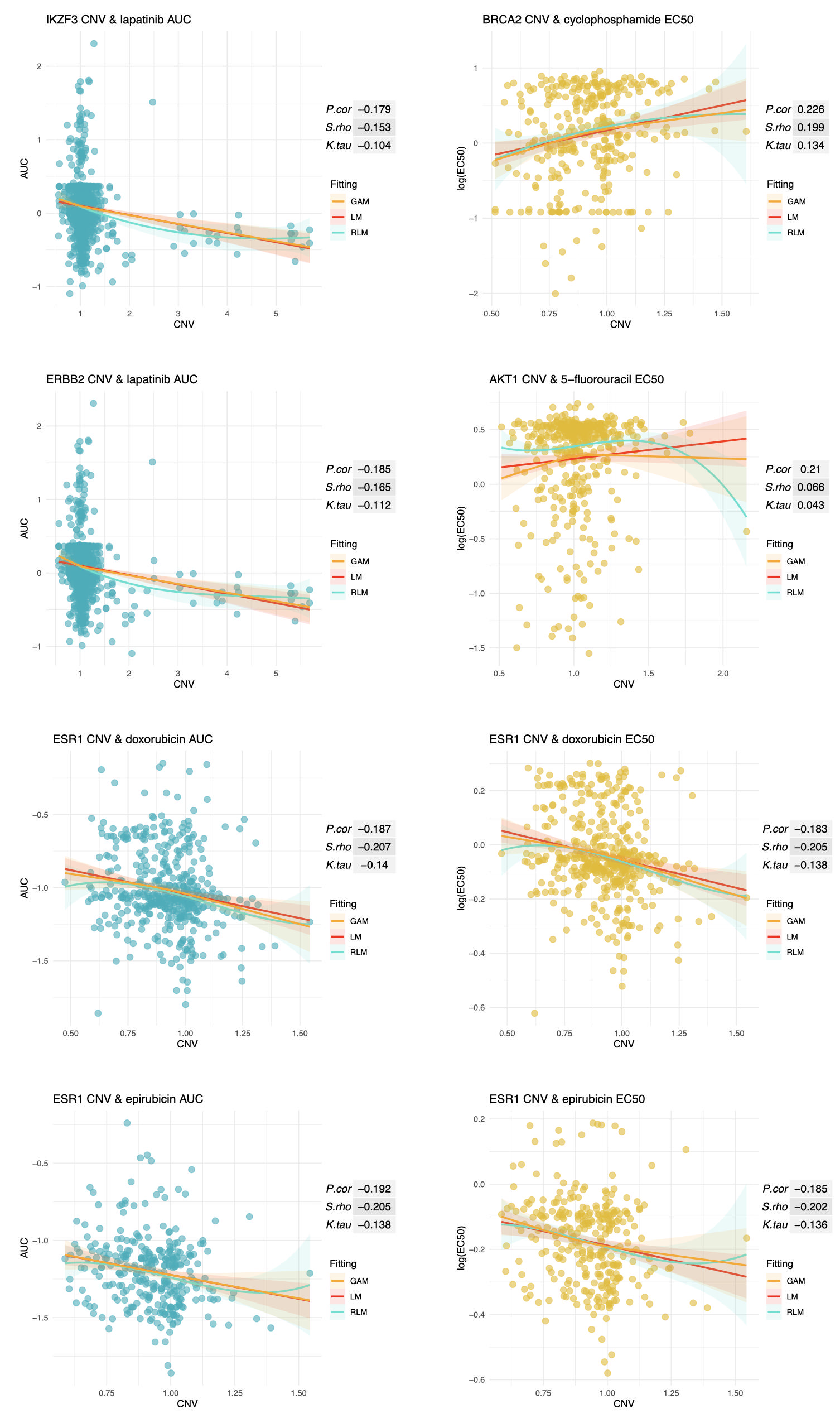

Supplement: S13 Fig — (TIFF) [file pone.0306343.s013.tiff]

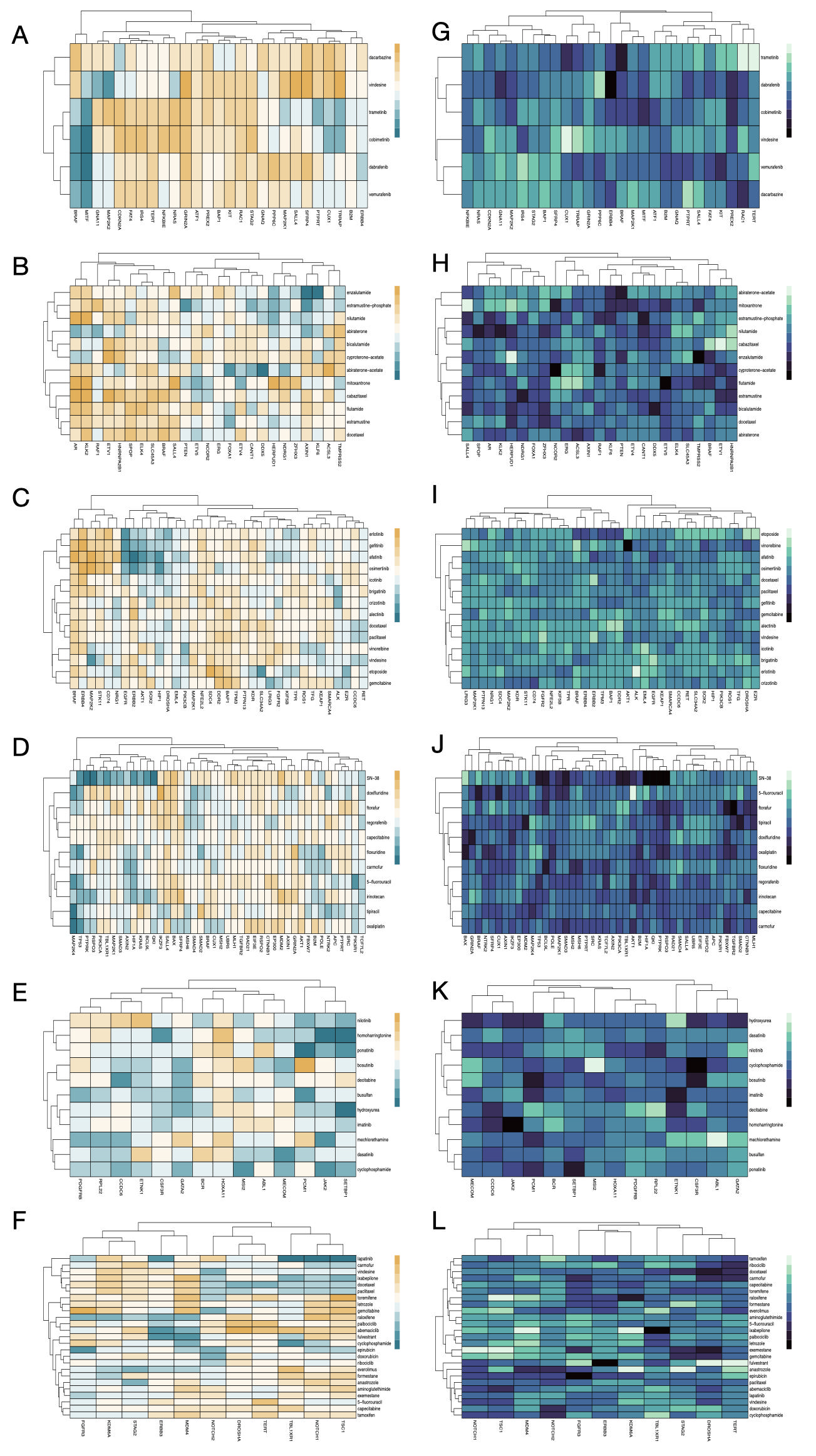

Supplement: S14 Fig — A: Gene-drug AUC map for SKCM. B: Gene-drug AUC map for PRAD. C: Gene-drug AUC map for NSCLC. D: Gene-drug AUC map for CRC. E: Gene-drug AUC map for CML. F: Gene-drug AUC map for of BRCA. G: Gene-drug EC50 map for SKCM. H: Gene-drug EC50 map for PRAD. I: Gene-drug EC50 map for NSCLC. J: Gene-drug EC50 map for CRC. K: Gene-drug EC50 map for CML. L: Gene-drug EC50 map for BRCA. (TIFF) [file pone.0306343.s014.tiff]

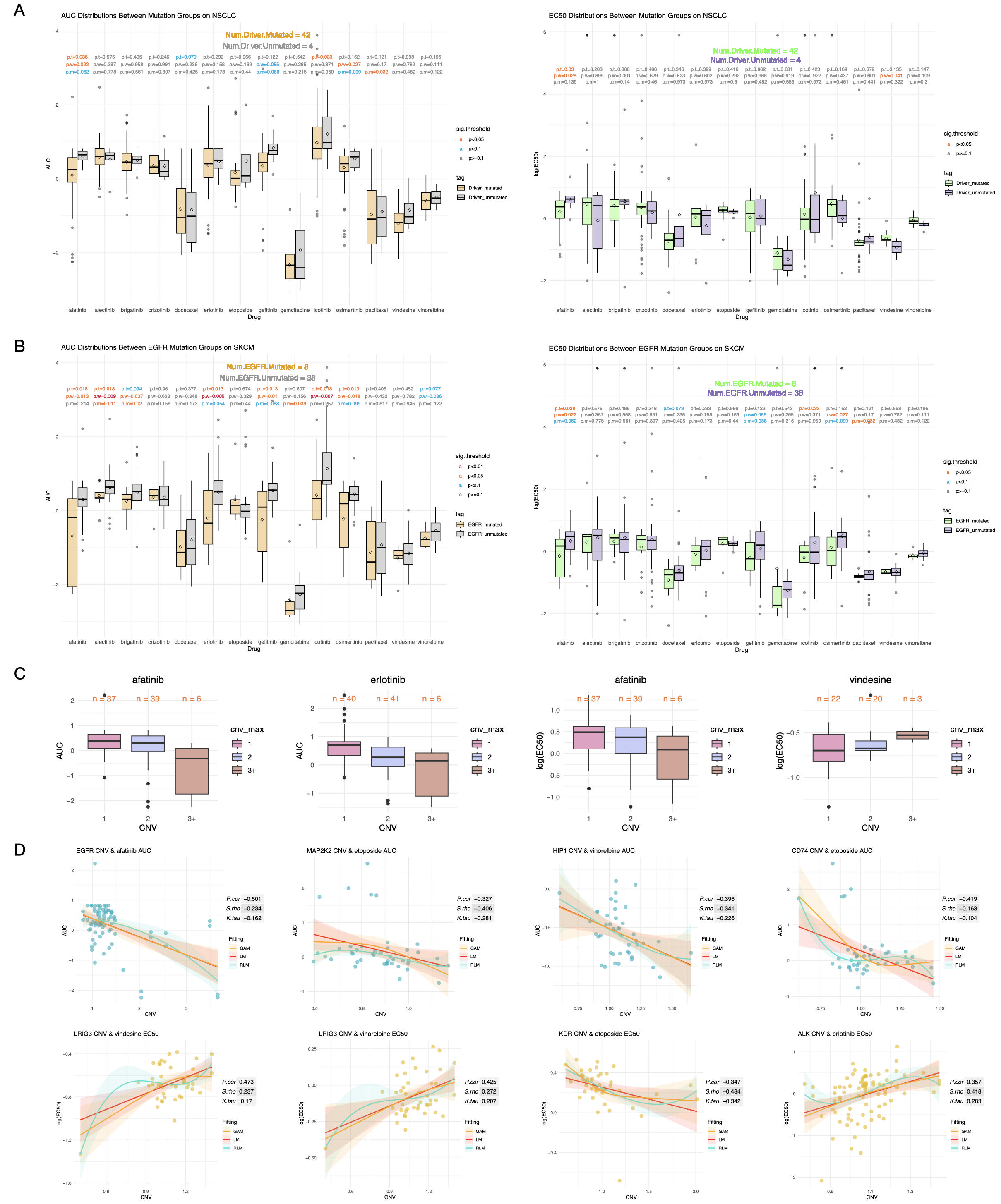

Supplement: S15 Fig — A: Box plots showing AUC distributions of NSCLC drugs with or without SNVs of NSCLC driver genes. B: Box plots showing EC50 distributions of NSCLC drugs with or without mutations of NSCLC driver genes. C: Box plots showing the correlation between the maximum CNV in driver genes and NSCLC drug response. D: Scatter plots showing the correlation of CNV and NSCLC drug response. (TIFF) [file pone.0306343.s015.tiff]

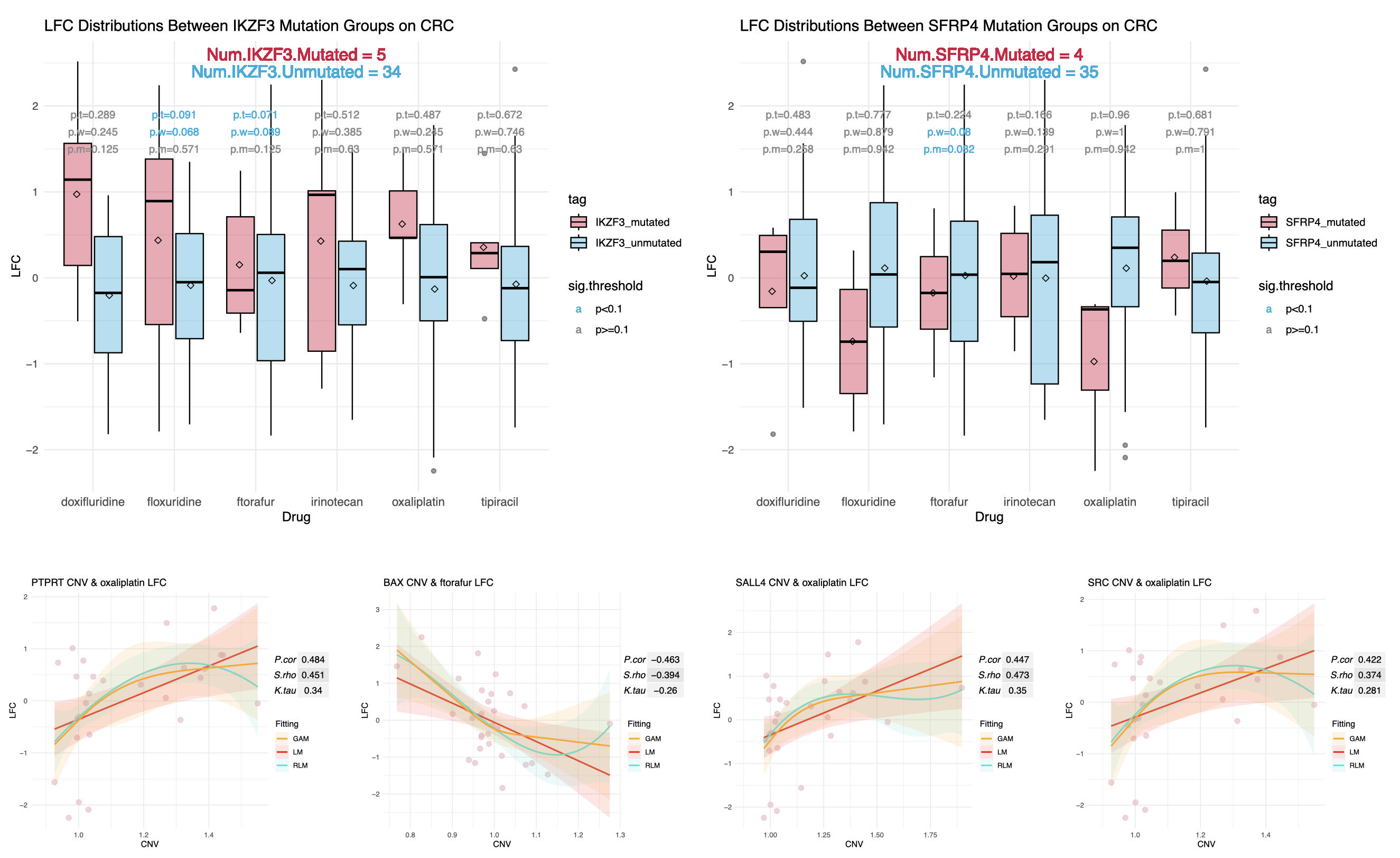

Supplement: S16 Fig — (TIFF) [file pone.0306343.s016.tiff]

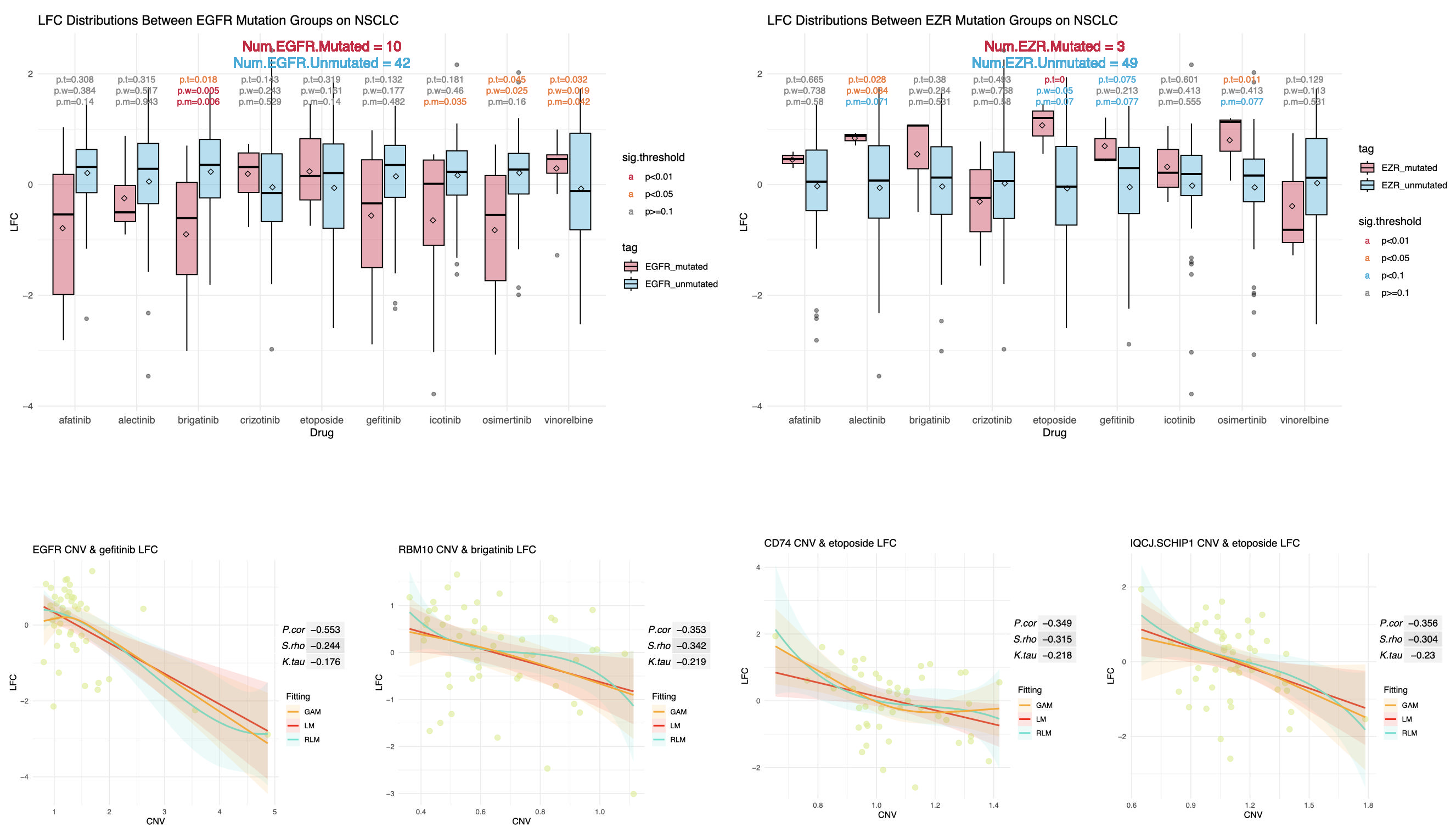

Supplement: S17 Fig — (TIFF) [file pone.0306343.s017.tiff]

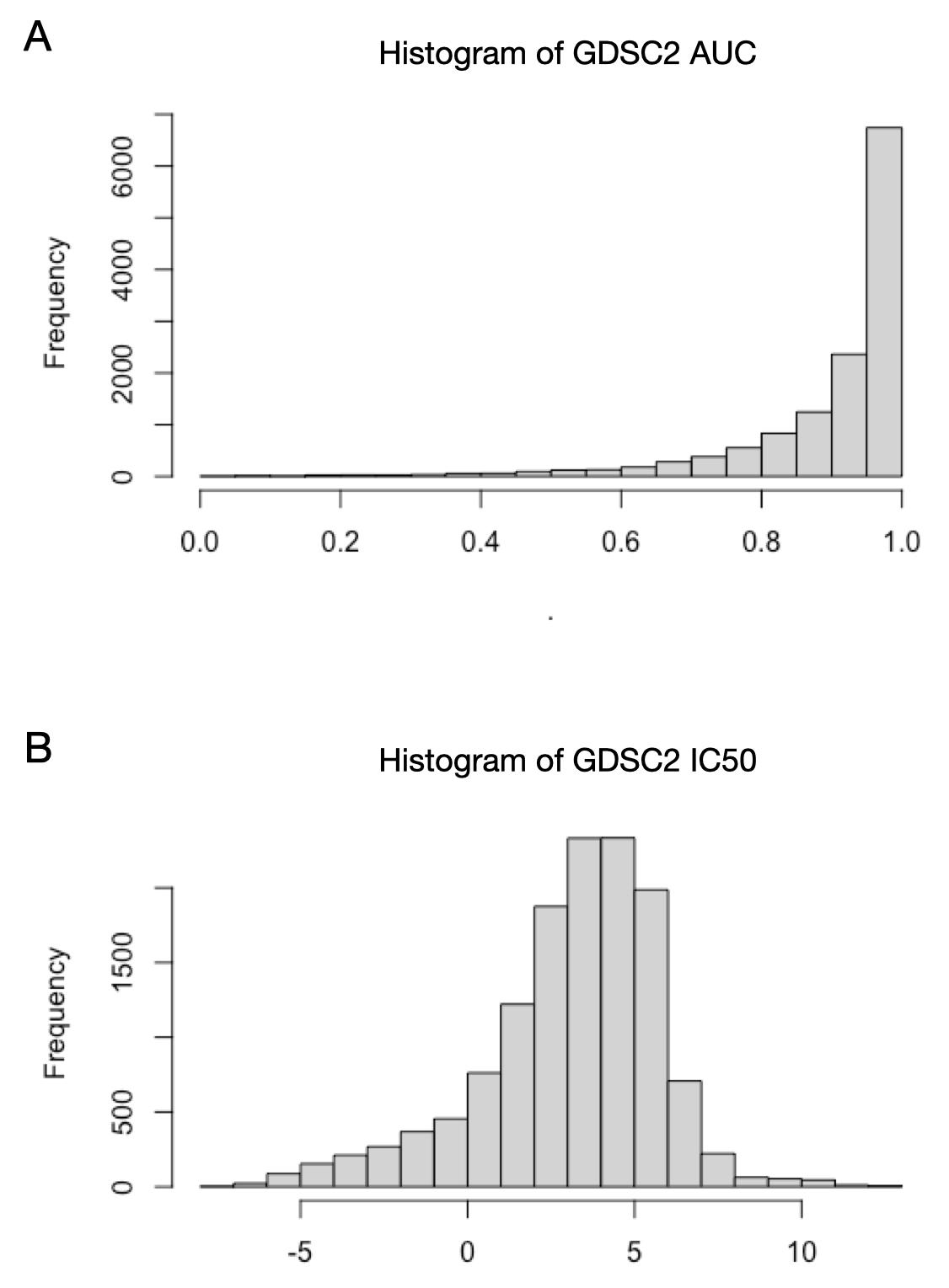

Supplement: S18 Fig — A: Histogram of GDSC2 AUC. B: Histogram of GDSC2 IC50. (TIFF) [file pone.0306343.s018.tiff]
